# Characterization of the Core Rumen Microbiome in Cattle during Transition from Forage to Concentrate as Well as during and after an Acidotic Challenge

**DOI:** 10.1371/journal.pone.0083424

**Published:** 2013-12-31

**Authors:** Renee M. Petri, Tyler Schwaiger, Greg B. Penner, Karen A. Beauchemin, Robert J. Forster, John J. McKinnon, Tim A. McAllister

**Affiliations:** 1 Lethbridge Research Centre, Agriculture and Agri-Food Canada, Lethbridge, Alberta, Canada; 2 Department of Animal and Poultry Science, University of Saskatchewan, Saskatoon, Saskatchewan, Canada; Northeast Agricultural University, China

## Abstract

This study investigated the effect of diet and host on the rumen bacterial microbiome and the impact of an acidotic challenge on its composition. Using parallel pyrosequencing of the V3 hypervariable region of 16S rRNA gene, solid and liquid associated bacterial communities of 8 heifers were profiled. Heifers were exclusively fed forage, before being transitioned to a concentrate diet, subjected to an acidotic challenge and allowed to recover. Samples of rumen digesta were collected when heifers were fed forage, mixed forage, high grain, during challenge (4 h and 12 h) and recovery. A total of 560,994 high-quality bacterial sequences were obtained from the solid and liquid digesta. Using cluster analysis, prominent bacterial populations differed (*P*≤0.10) in solid and liquid fractions between forage and grain diets. Differences among hosts and diets were not revealed by DGGE, but real time qPCR showed that several bacteria taxon were impacted by changes in diet, with the exception of *Streptococcus bovis*. Analysis of the core rumen microbiome identified 32 OTU's representing 10 distinct bacterial taxa including Bacteroidetes (32.8%), Firmicutes (43.2%) and Proteobacteria (14.3%). Diversity of OTUs was highest with forage with 38 unique OTUs identified as compared to only 11 with the high grain diet. Comparison of the microbial profiles of clincial vs. subclinical acidotic heifers found a increases in the relative abundances of *Acetitomaculum*, *Lactobacillus*, *Prevotella*, and *Streptococcus*. Increases in *Streptococcus* and *Lactobacillus* likely reflect the tolerance of these species to low pH and their ability to proliferate on surplus fermentable carbohydrate. The acetogen, *Acetitomaculum* may thereforeplay a role in the conversion of lactate to acetate in acidotic animals. Further profiling of the bacterial populations associated with subclinical and clinical acidosis could establish a microbial fingerprint for these disorders and provide insight into whether there are causative microbial populations that could potentially be therapeutically manipulated.

## Introduction

The rumen microbiome is an extremely diverse and well-studied microbial ecosystem [1], [2] and acidosis is among the most researched rumen conditions owing to its negative impact on cattle production [3]. Most commonly, acidosis results from fermentation of starch leading to a rapid increase in the concentrations of short chain volatile fatty acids in the rumen and a precipitous drop in pH [4]. Classical microbial techniques using anaerobic fermentation analysis have shown that this rapid accumulation of acid within this environment alters the activity and abundance of many bacterial species [5], [6].

Molecular profiling of microbial communities has shown that traditional culture techniques underestimate bacterial diversity in the rumen [7], [8]. The use of DGGE, qPCR and 454 sequencing has shown that there are changes in the rumen bacterial populations that occur with dietary change [8], [9], [10]. However, changes in rumen microbial populations as a result of acidotic inducing diets within individual animals has not been well elucidated. Next generation sequencing of the human gut microbiome suggests that bacterial community composition is surprisingly stable within individuals [11]. However, elements of each host's microbiome appear unique [12], [13], and these rare species may influence the risk or occurrence of disease [14]. Despite the differences in gastrointestinal physiology, it can be assumed that many of the concepts from the human gut microbiome are also likely relevant to the intestinal microbiome of other mammals including cattle. Therefore, it is likely that similar to the human gut [15], there is a rumen ‘core’ microbiome in cattle that remains stable regardless of differences in diet or host genetics [10], [16]. If this were proven true, then identification of deviations from this core microbiome may also be indicative of the risk or occurrence of disease [15], [17] Given that many bacteria within the rumen only utilize specific substrates, changing the composition of the diet is likely to have the greatest impact on the composition of the rumen core microbiome [18]. Classical rumen microbiology has shown that microbial populations shift substantially during clinical acidosis, with cellulolytic bacteria declining and acid tolerant bacteria such as *Streptococcus* and *Lactobacillus* spp. proliferating [19]. Therefore, the objective of this study was to examine populations during transition from forage to concentrate diets, as well as during and after an imposed episode of acidosis. It was hypothesized that the diversity of bacteria in the rumen would decrease as the forage to concentrate ratio of the diet decreased and acidosis occurred.

## Materials and Methods

### Animals and sampling

Samples for this study were collected similar to Petri *et al.* [10] deriving data from an investigation that used 8 Angus heifers to study the impact of an acidotic challenge on rumen function [20]. Angus heifers were cared for in accordance with the guidelines of the Canadian Council of Animal Care [21] and the protocol was reviewed and approved by the Lethbridge Research Centre Animal Care Committee. Heifers (BW: 308 kg±35 SD) were raised exclusively on forage diets and upon initiation of the study were randomly assigned to one of four groups that progressed through 5 dietary treatments over 11 wk. Each group was progressed through the diet treatments with an offset of 3 wk due to the intensity of sampling procedures. Heifers were fed the same forage diet with a mineral supplement for 3 wk prior and sampled on the first day of the experiment (d. −1). Subsequently, they were abruptly transitioned to a mixed forage – concentrate diet consisting of 60% forage and 30% barley grain and 10% supplement and adapted to this diet for two wk prior to the second sampling (d. 15). After sampling, heifers were transitioned over 20 d to a high grain diet (high grain) consisting of 81% barley grain and 9% forage with 10% concentrate supplement. After the transition animals were maintained on this diet for 30 d prior to the next sample collection (d. 65). One week later they were subjected to an acidotic challenge (d.72). The challenge was administered by restricting intake to 50% of ad libitum feed intake as measured over the 4 d prior to the challenge. After 24 h of restriction, a single pulse dose of ground barley grain was administered into the rumen via the cannula. Heifers in Group 1 received a dose equal to 20% of ad libitum intake, whereas heifers in Groups 2, 3, and 4 received a dose equal to 10% of ad libitum intake. For all groups, the dose of barley grain was calculated as a proportion of ad libitum intake with consideration for body weight. One hour after the intraruminal dose of grain, heifers received their regular allotment of the high concentrate diet. To monitor the severity of the challenge, the pH of rumen fluid from the ventral sac was measured at 2 h intervals for the first 12 h and at 4 h intervals for a further 12 h. If ruminal pH was below 4.2, pH was measured again 1 h later and if it remained below 4.2, 250 g of sodium bicarbonate was dosed into the rumen in accordance with the animal care protocol. Rumen contents were also collected 1 wk after challenge (d. 79) to monitor the degree to which bacterial populations reverted to pre-challenge conditions.

### Rumen sampling

Sampling of rumen contents for bacterial analysis occurred at 4 h post-feeding on the collection day for each dietary treatment, except on the day of the acidotic challenge when an additional sample was collected 12 h post-feeding. Whole rumen contents were collected into a wide mouth 500 mL bottle (Sigma-Aldrich Canada Ltd., Oakville, ON, Canada) from five sites (cranial, caudal, dorsal, caudal ventral) within the rumen. The bottle was sealed and samples were immediately transported to the laboratory for processing.

Rumen pH was continuously monitored during the acidotic challenge and recovery period using the Lethbridge Research Center Ruminal pH Measurement System (LRCpH; Dascor, Escondido, CA) [22]. Daily ruminal pH data were summarized as minimum pH, mean pH, maximum pH as well as duration and area below the pH thresholds 5.8, 5.5 and 5.2 [22]. These data have been previously reported by Petri *et al.* [10].

### Bacterial DNA extraction and pyrosequencing

Rumen samples were processed immediately upon collection as described previously [23]. Briefly, rumen liquid samples were obtained by mixing the collected rumen contents in the centrifuge bottle, placing those contents into a heavy walled 250 mL beaker and separating the particulate and liquid using a Bodum coffee filter plunger (Bodum Inc., Triengen, Switzerland). Aliquots of fluid digesta (5 mL) were place in aluminum foil dishes and flash-frozen in liquid nitrogen and stored at −80°C. Solid rumen content samples were collected by removing the remainder of the liquid contents with the Bodum coffee filter and then flash freezing subsamples (∼5 g) in liquid nitrogen that were stored at −80°C until further processing.

Genomic DNA was extracted as described by Kong *et al.* [24]. All samples were individually ground to a fine powder in liquid nitrogen using a mortar and pestle, combined with proteinase K (1 mg/mL; Sigma-Aldrich Canada Ltd. Oakville, Ontario, Canada) and further ground in liquid nitrogen for 5 min (model RM100 grinder, Retsch GmbH, Haan, Germany). Samples of liquid and solid digesta were processed on separate days to avoid cross contamination. Each sample was mixed with ∼100 mL of liquid nitrogen and transferred to a 200 mL wide-mouth centrifuge bottle and incubated for 40 min at 50°C in a water bath to thaw the samples. After incubation, 15 mL of sample was transferred into a 50 mL polycarbonate tube (SS34; Fischer Scientific Ltd, Nepean, Ontario, Canada) containing 1.5 mL of 20% vol/vol SDS (Sigma-Aldrich Canada Ltd. Oakville, Ontario, Canada). The resultant mixture was incubated for 45 min at 65°C in a water bath and then centrifuged at 10,000× g for 10 min. Three equal volumes of supernatant were combined with preheated (65°C) 2% agarose mixture (Sigma-Aldrich Canada Ltd., Oakville, Ontario). The suspension was gently inverted to create a homogenous mixture and transferred to petri dishes (15 mm H; Fischer Scientific Ltd, Nepean, Ontario, Canada) and allowed to cool at room temperature. Once set (1 h) agarose samples were cut into strips (1 cm wide) and washed in 10 volumes of TE buffer (10∶2 of 1M Tris-HCl to 0.5M EDTA) for 16 h. Agar (200 mg) containing cleaned sample DNA were distributed between triplicate 1.5 mL snap cap tubes (Fischer Scientific Ltd, Nepean, Ontario, Canada) and placed in −80°C for 1 h. Frozen samples were “freeze-squeezed” [25] by centrifuging at 10,000× g for 10 min to extract the DNA fragments from the agar. The resulting supernatant of TE buffer containing bacterial DNA was transferred to a new 1.5 mL tube. Samples were then refrozen at -80°C for 1 h and centrifuged once again. Supernatants were combined from the repeat centrifugation and all samples were stored at 4°C prior to analysis. DNA from each sample was quantified using fluorometric dsDNA using picogreen dye (Invitrogen, Life Technologies Inc., Burlington, ON, Canada) and measured with a synergy HT plate reader (BioTek U.S. Ltd, Winooski, VT, United States). Subsequently, individual genomic DNA samples for all treatments were diluted to a concentration of 20 ng µL^−1^ in TE buffer. One 20 µL aliquot of each sample for a total of 36 genomic DNA samples (forage n = 5; mixed n = 8; high grain n = 7; acidotic challenge n = 8; challenge recovery n = 8) were sent to the Research and Testing Laboratory (Lubbock, TX, USA) for pyrosequencing using a 454 GS FLX Titanium Sequencing System (454 Life Sciences, a Roche company, Branford, CT, USA). Those samples that were unable to meet the requirement of 20 ng µL^−1^ in 20 µL of TE buffer were not sent for sequencing. Pyrosequencing targeted the V1 to V3 hypervariable region of the 16S rRNA gene as described by Dowd *et al.* [26].

### PCR-DGGE analysis

Extracted, diluted DNA (60 ng) from each sample was added as template to amplify the V3 region of the 16S rRNA gene for PCR-DGGE analysis in a 25-µl reaction. Amplification was performed using Qiagen HotStar Plus Master Mix Kit (Qiagen) and 50 nM of forward and reverse primers (341f with GC-Clamp:CGCCCGCCGCGC-GCGGCGGGCGGGGCGGGGGCACGGGGGGCCTACGGGAGGCAGCAG and 534r: ATTACCGCGGCTGCTGG) developed by Muyzer *et al.* [27] as previously reported [9]. Polymerase chain reaction conditions were 95°C for 5 min, 94°C for 30 s, temperature gradient decreasing from 65°C to 55°C by 0.5°C each cycle for 30 s, 72°C for 1 min for 20 cycles, followed by 94°C for 30 s, 56°C for 30 s, 72°C for 1 min for 10 cycles and 72°C for 10 min for final elongation. Amplified DNA was assessed for quality using gel electrophoresis and quantified using flurospectrophotometry by measuring the *A*_260/280_ (ND-3300 Nanodrop, Wilmington, DE, U.S.A). Amplified DNA was then normalized to 100 ng µL^−1^ and 4 µL DNA along with 4 µL of 2× loaded dye (0.05% bromophenol blue, 0.05% xylene cyanol, 70% glycerol w/v in H_2_O, pH 8.0) were placed in each lane on 8% acrylamide gels with a 45–60% denaturing gradient of urea and formamide. Electrophoresis was performed at 60°C and 40 V for 20 h. Three lanes on each gel were loaded with DGGE Marker II (Wako, Nippon Gene, Japan) to provide both an internal and external marker. Gels were stained with SybrGold Nucleic Acid Gel Stain (Invitrogen, Life Technologies Corp., Carlsbad, CA, U.S.A) according to manufacturer's instructions and photographed by UV transillumination.

### Real-time PCR

Quantitative analysis was performed with the ABI PRISM 7700 Sequence Detection System (AB Applied Biosystems, Life Technologies Corp.), to quantify the relative abundance of 16S rRNA genes of several groups: Genus *Ruminococcus*, genus *Prevotella*, *Selenomonas ruminantium*, *Fibrobacter succinogenes*, *Megasphaera elsdenii*, and *Streptococcus* spp. were all measured as a percentage of total bacterial 16S rRNA using previously reported primers [9]. The quantification of DNA for each bacterial species in rumen contents was performed using a Quantifast Kit (Qiagen) with SYBR green chemistry. Standards and samples were assayed in 25 µl reaction mixture containing 15 µl of master mix, 8 µl of nuclease-free water and 2 µl of DNA template. The amplification programs was: 95°C for 5 min, 95°C for 10 s and a 30 s annealing/elongation for 40 cycles [9]. The melting curve of PCR products was monitored by slow heating with an increment of 0.1°C s^−1^ from 60−95°C with fluorescence measured at 0.1°C intervals to confirm specificity of amplification. A standard curve for each bacterial species was constructed by using plasmid DNA containing 16S rRNA inserts of DNA purified from a pure culture of the target species [28]. *Ruminococcus* plasmid DNA was used as a standard template for universal bacteria primers. Plasmid DNA was quantified and subject to seven sequential ten-fold dilutions with each analyzed in duplicate. A linear relationship was observed between the threshold cycle (C_t_) and log of DNA concentration when each primer pair was tested against purified DNA from its target taxon. Each sample was run in triplicate and the PCR reaction cycle at which the reaction exceeded this was identified as the C_t_. The copy number of total bacteria and each target species was determined by relating the C_t_ values to standard curves using the following equation:

DNA (number of molecules)  =  (6.02×10^23^ (molecules/mol) × DNA amount (ng))/(DNA plasmid-insert length (bp) ×6.6×10^11^(ng mol^−1^ bp^−1^))

Amplification products were verified by horizontal gel electrophoresis of a 5 µl aliquot in a 1% agarose gel in Tris-Acetate-EDTA (40 mM Tris acetate, 1 mM EDTA; pH 8.5), followed by ethidium bromide staining and visualisation under UV light. A 1 kb Ladder (Quickload, New England Biolabs Ltd., Pickering, ON, Canada) was included on each gel to enable confirmation of the size of the amplified product.

To minimize errors of absolute quantification of DNA from rumen samples, relative quantification methods were used. In relative quantification, amplification is expressed relative to the amplification of reference primers utilizing experimentally derived amplification efficiency [28], [29]. The proportion of each species was estimated by dividing the copy number of 16S rRNA gene sequences of the targeted species by the 16S rRNA gene sequences amplified with a reference primer set [19]. A non-degenerate, domain-level primer set that amplified all bacterial species was used as the reference primer set [9].

### Pyrosequence analysis

**Figure 1:**
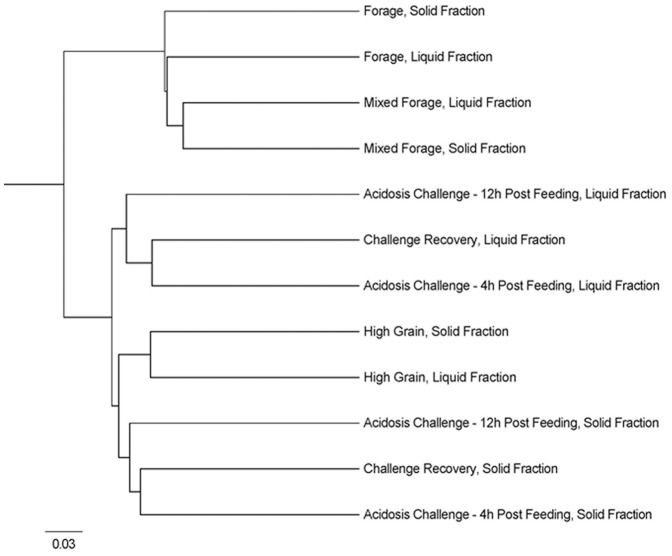
Cluster analysis of dietary treatments created using Jaccard analysis to show dissimilarity between dietary treatments. Comparison of the unique OTU's found in each dietary treatment (forage, mixed forage, high grain, acidotic challenge 4 h post-feeding, acidotic challenge 12 h post-feeding, recovery), separated based on digesta fraction (solid and liquid).Clusters were created using Jaccard analysis to show dissimilarity among treatments. OTUs are estimated at a 10%.

Pyrosequencing analysis of the V1−V3 region of 16S rRNA on 36 samples yielded 613,689 raw reads. Reads with an average quality score of less than 35, as well as homopolymers greater than eight bases, and sequences with one or more ambiguous bases were removed from the data set in accordance with the 454 pipeline standard operating procedure from Scholss et al. [30], [31] Sequences were then aligned against the SILVA alignment database (version 108) using the Mothur program to define operational taxonomic units (OTUs) [30]. Sequences that did not span the longest alignment region were also removed from the dataset. Sequences were trimmed so that reads overlapped in the same alignment space [30], [31] resulting in read lengths ranging from 167 to 349 bps. Pyrosequencing base call errors were minimized using the pre-cluster algorithm in Mothur [32], whereby rare sequences that were highly similar to abundant sequences were re-classified as their abundant homologue. Chimeras were removed from the samples using the sequence collection (UCHIME) as its own reference database [31], [33]. A distance matrix was constructed using the average neighbor algorithm at 0.03 (equivalent to species), 0.05 (genus), 0.25 (phylum) phylogenetic distances. Pairwise distances between aligned sequences were calculated and at a 97% similarity cutoff sequences were clustered into unique OTUs. In total, there were 560,994 high quality reads with an average of 3,937±921 reads and 128±23 unique OTUs per individual sample. Mothur was also used to calculate the coverage (rarefaction curves; Figure S1), the number of species in a sample (species richness; Chao1) and abundance-based coverage, as well as the number of equally-abundant species (species diversity) with Shannon-Wiener and Simpsons indices (Table 1). A dendrogram (Figure 1) based on treatment differences was generated using the Jaccard index [30]. Sequences and OTUs were classified using the Mothur program against the Silva reference v108 database [34] set to name uncultured clusters after the most recently deposited clone sequence. All sequences generated have been submitted to the European Nucleotide Archive (ERS361511 to ERS361603). Calculation of the percentage of sequences within a taxonomic classification at the genus and species level was performed using a custom summation script.

**Table 1:** Average of individual heifers, for all samples collected over the duration of the study, for unique OTUs, richness estimates, and diversity indices within the liquid and solid rumen digesta.

	Individual heifers									
	7	41	43	143	153	156	315	346	SEM^1^	*P*-value
Number of Sequences	4450abc	5521c	5243bc	3698abc	3868abc	3296a	3436ab	5427c	409	<0.001
Coverage (%)	91.9a	99.2b	99.1b	91.9a	94.9a	93.0a	94.2a	99.0b	1.8	0.01
Total # of Unique OTUs	107abc	152d	151d	130abcd	141bcd	94a	103ab	147cd	9	<0.001
Richness Estimate										
Chao1^2^	146abc	195c	198c	175abc	184bc	124a	134ab	197c	12	<0.001
ACE	163ab	196b	200b	180ab	195b	131a	138a	204b	12	<0.001
Diversity Indices										
Shannon-Weiner	2.68ab	3.03bc	2.83abc	3.00bc	3.20c	2.76abc	2.50a	2.93abc	0.10	<0.001
Simpson's	0.16ab	0.14ab	0.18b	0.10a	0.10a	0.15ab	0.20b	0.16ab	0.02	<0.001

^1^ SEM: standard error of the mean.

^2^ ACE: abundance coverage estimator.

Letters which differ in the same row indicate a significant difference between values within the row.

### Statistical analysis

Analysis of PCR-DGGE band patterns was accomplished using BIONUMERICS software (Version 5.1, Applied Maths, Inc., Austin, TX, USA) to create similarity matrices to identify differences in community populations among treatments and individual animals. Bands were visually selected based on peak height. Using average Dice's similarity coefficient (D_sc_) index, with an optimization of 1.0% and a tolerance of 1.0%, clustering was carried out using the unweighted pair group method with arithmetic means (UPGMA). Read number, sample coverage, unique OTUs, sample richness (Chao1 and ACE) and sample diversity (Shannon-Weiner and Simpson's indices) were compared with one-way ANOVA using the Proc Mixed procedure of SAS (version 9.1.3; SAS Institute Inc., Cary, NC, USA). Using the same procedure, qPCR relative quantification and rumen fermentation variables including VFA and pH were analyzed for effect of diet, heifer and the interaction between diet and heifer. Percent taxonomic data were log-transformed prior to analysis. Samples taken at 4 h and 12 h post acidotic challenge were treated as independent samples. Means were separated using Tukey's honest significant difference (HSD). All pH variables were additionally analyzed in a pairwise correlation to all unique OTUs using Proc Corr model of SAS. Significance was declared at *P*≤0.05 with trends indicated at *P*≤0.10.

## Results

### Bacterial community composition, abundance and occurrence

**Figure 2:**
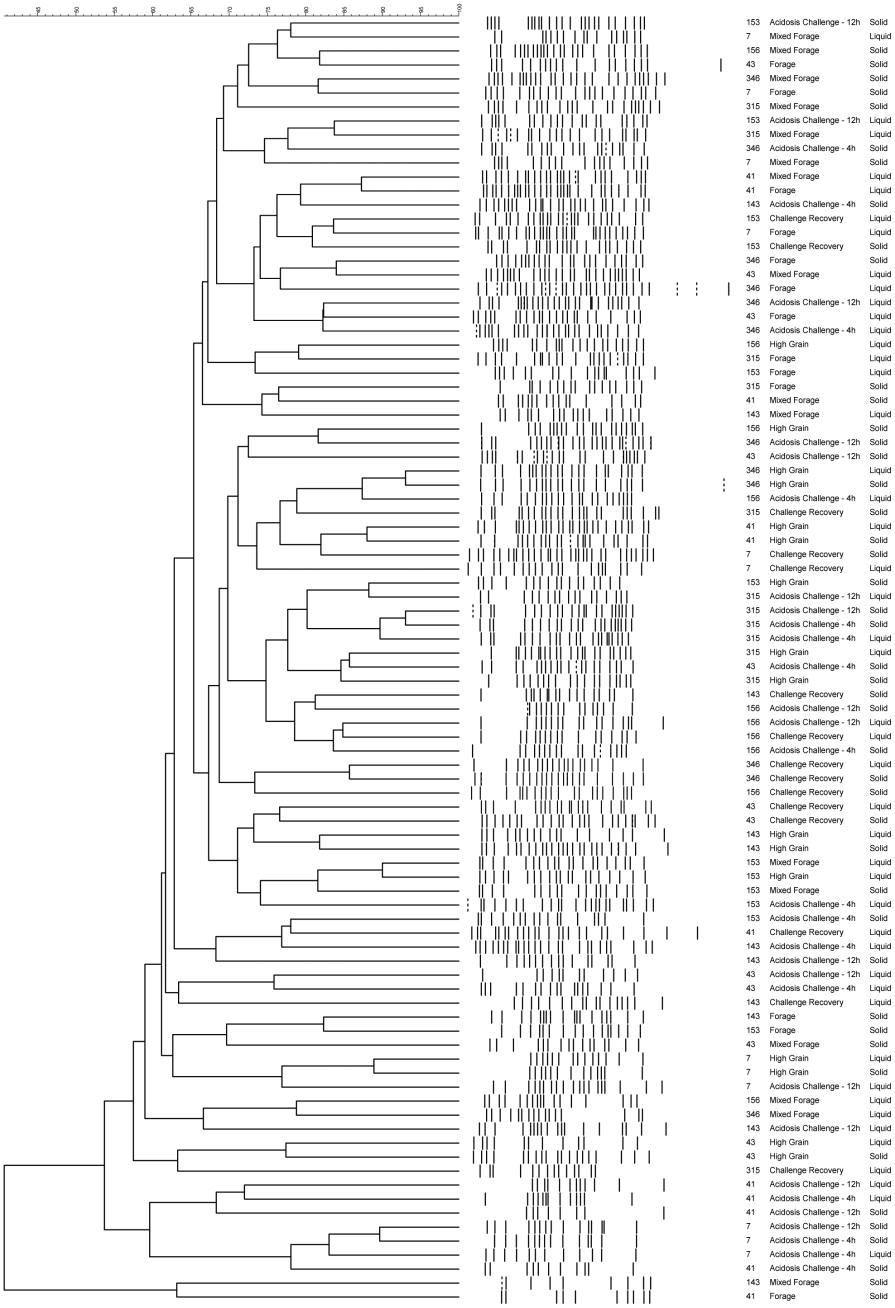
Dendrogram of PCR-DGGE analysis of rumen samples from cattle fed a progression of dietary treatments. Cluster analysis using Dice (Opt:1.0%) (Tol 1.0%-1.0%) and UPGMA was performed on PCR-DGGE analysis fingerprints from the liquid and solid fractions of the rumen digesta, from all heifers fed a progression of dietary treatments :forage, mixed forage, high grain, acidotic challenge (4 h and 12 h post-feeding) and recovery.

Using PCR-DGGE to compare the overall diversity of each sample, cluster analysis showed no significant clustering of profiles based on individual animal, dietary treatment or fraction of digesta (Figure 2). Cluster analysis performed on 454 sequence data showed that, while not significantly different, dietary treatments for forage and mixed forage diets clustered separately from diets containing predominantly grain (high grain, acidosis challenge and challenge recovery). Real-time qPCR analysis of six different bacterial targets within each of the six samples collected (i.e., forage (d. −1), mixed forage (d. 15), high grain (d. 65), 4 and 12 h post-acidotic challenge (d. 72) and recovery (d. 790) as well as between solid and liquid digesta are shown in Table 2. All of the bacterial targets, except for *Streptococcus bovis* were found to be affected by dietary treatment. *Ruminococcus* spp. and *Fibrobacter succinogenes* accounted for a large percentage of the total bacteria in the mixed forage diet (18.09 and 3.64%, respectively) and contributed the least to the high grain diet (5.70 and 1.60%, respectively). *Prevotella* was most prominent during the acidotic challenge and lowest in those heifers fed forage (Table 2). *Selenomonas ruminantium* and *Megasphaera elsdenii* accounted for the smallest proportion of the bacterial population in heifers fed forage (1.12 and 0.01%, respectively). *M. elsdenii* was present in the highest density in heifers, 12 h after acidotic challenge and declined during recovery, whereas *S. ruminantium* increased. None of the qPCR targeted bacteria differed between the solid and liquid digesta, excepting *F. succinogenes*, which was 1.23% higher in the solid-digesta (Table 2).

**Table 2:** Effect of dietary treatment and digesta fraction on the percent of total enumerated eubacteria 16S rRNA genes of dominant rumen bacterial species using quantitative real-time PCR.

	Dietary treatment^1^								Digesta fraction^1^			
	Forage	Mixed forage	High grain	Acidosis challenge		Challenge recovery						
Bacteria				4 h PF^2^	12 h PF		SEM	*P*-value	Liquid	Solid	SEM	*P*-value
*Ruminococcus* spp.	8.01a	18.09b	5.70a	10.02a	6.75a	12.75a	1.820	<0.001	9.91	10.63	1.025	0.572
*Fibrobacter succinogenes*	2.86b	3.64b	0.16a	0.91a	0.57a	0.64a	0.489	<0.001	0.82a	2.05b	0.282	0.004
*Prevotella* spp.	2.86a	4.47ab	7.75bc	10.78c	7.22abc	8.26bc	1.120	0.002	7.67	6.29	0.644	0.118
*Selenomonas ruminantium*	1.12a	1.75ab	6.44cd	4.97bc	4.48bc	8.17d	0.725	<0.001	4.78	4.34	0.725	0.419
*Megasphaera elsdenii*	0.01a	0.18ab	0.43ab	0.50ab	0.69b	0.20ab	0.159	0.049	0.31	0.37	0.092	0.666
*Streptococcus bovis*	0.07	0.08	0.00	0.20	0.92	0.02	0.490	0.760	0.02	0.42	0.281	0.342

^1^ Factorial analysis was performed however none of the interactions were significant with the exception of Animal×Treatment interactions for *Selenomonas ruminantium* and *Megasphaera elsdenii* (*P*<0.05). Significant Treatment × Fraction interactions for *Selenomonas ruminantium* (*P*<0.05).

^2^ PF: post-feeding.

Letters which differ in the same row indicate a significant difference between values within the row.

**Figure 3:**
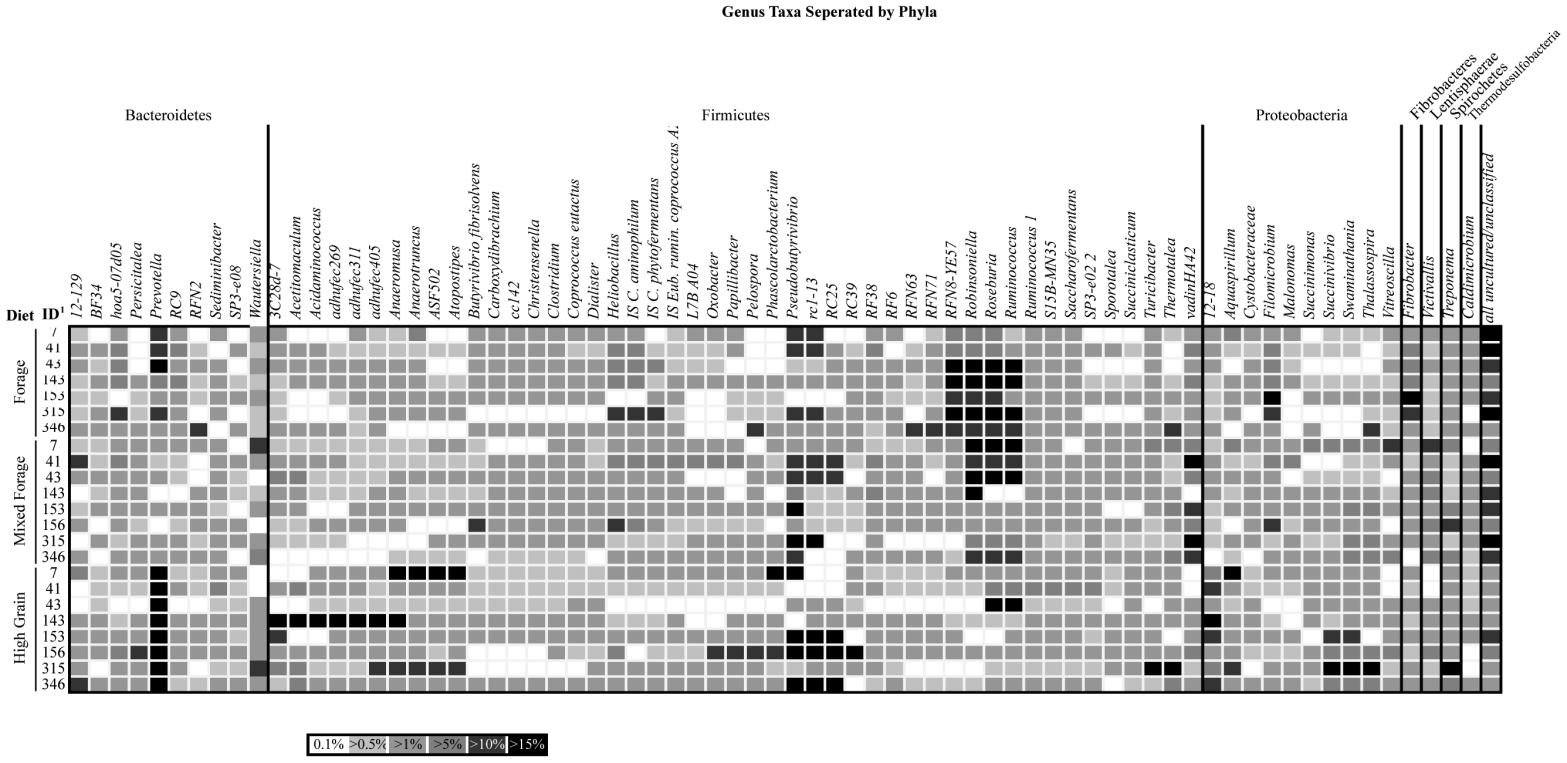
Heat map of the relative percent abundance of all genera for each individual heifer. This diagram depicts the change in relative percent abundance of all genera for each individual heifer (indicated by ID number) for each of the three dietary regimes: forage, mixed forage and high grain. Percent abundance increases with the darkness of the corresponding square from ≤1.0% to >15% of the total rumen population as averaged between the liquid and solid fractions.

Rarefaction curves determined for each of the dietary treatments showed a higher diversity of OTU's in forage and mixed forage diets compared to those that were predominantly high in grains (Figure S1). On average, 21,000 to 36,000 sequences were determined for each of the dietary treatments. However, a plateau was not reached for any of the dietary treatments indicating that additional sequencing would be necessary to fully describe rumen bacterial communties under these conditions. The relative abundance of all genera was compared among heifers at each of the five sampling times (day 1, 15, 65, 72, 89) using a heat map (Figure 3). This analysis found 72 distinct genera that varied in abundance among heifers, or across diets. The average number of sequences per animal within the digesta fraction ranged from 3260 in liquid from a heifer fed forage to 6832 in the liquid from a heifer 4 h post-challenge. The average number of sequences per heifer between solid and liquid digesta were similar (data not shown). Percent abundance data identified 59 genera that were affected either by sampling time, digesta fraction or their interaction. A total of 35 genera including members of the Bacteriodetes phylum (*JW17*, *RC9* and *Prevotella*), the Firmicutes phylum (*Acidaminococcus*, *Blautia*, *Lactobacillus*, *Marvinbryantia*, *Papillibacter*, *RFN71*, *RFN8-YE57*, *Roseburia*, *Saccharofermentans*, *Selenomonas*, *Solobacterium*, *Sporobacterium*, *Sporotalea*, *Streptococcus* and *vadinHA42*), the Proteobacteria phylum (–, *Pannonibacter*, *Ruminobacter*, *Succinivibrio*, *Thalassospira*) as well as *Fibrobacteres* (phylum *Fibrobacter*), *Treponema* (phylum *Spirochaetae*) and a large number of unidentified/uncultured taxa were found to be affected by diet or acidotic challenge. Of these, a significant effect of host was found for *12*–*18*, *Acidaminococcus*, *Blautia*, *Papillibacter*, *Prevotella*, *RC9*, *Roseburia*, *Selenomonas*, *Solobacterium*, *Succinivibrio*, *Thalassospira*, and *Treponema*. When comparing percent abundance between solid and liquid digesta, 9 of 13 genera including *Atopostipes*, *Fibrobacter*, *Selenomonas*, *Sporotalea*, *Treponema*, *Wautersiella* and *Xylanibacter* were found to be highest in solid digesta.

### Core microbiome

**Figure 4:**
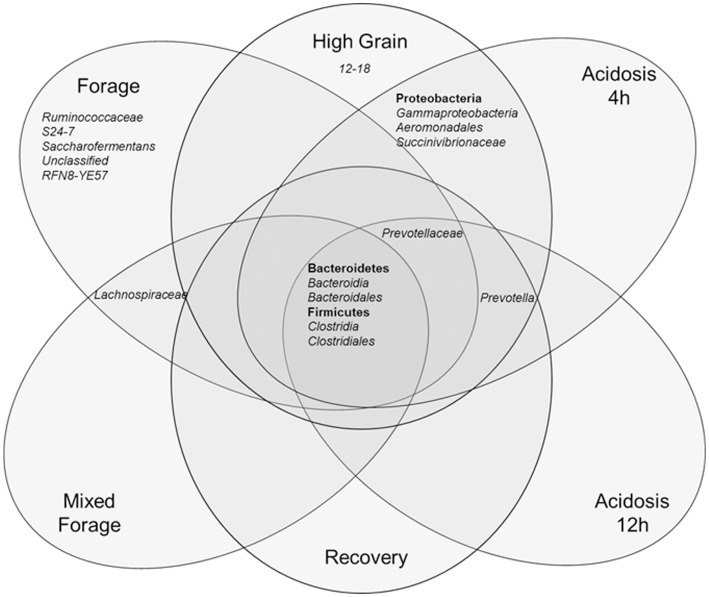
Venn diagram of the rumen core microbiomes. The 6 circle venn diagram shows the rumen core microbiomes as determined by only those taxa which were ubiquitous for the solid and liquid-digesta and for all heifers. Each circle represents a diet, bacterial taxa within overlapping areas were common to the corresponding diets.

**Figure 5:**
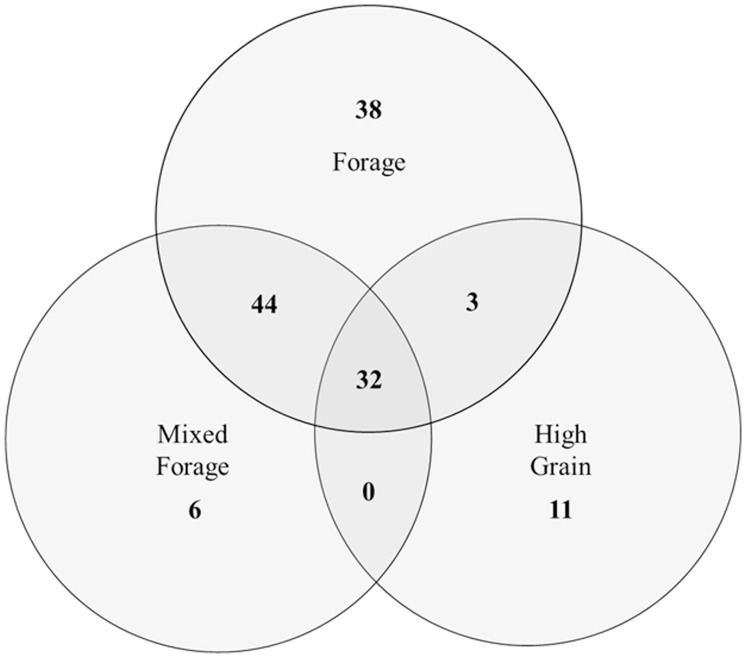
Venn diagram of the dominant OTU's for each diet. The three basal diets used in this study each showed a number of unique OTU's. Each circle represents each of the major diets (forage, mixed forage, high grain) with numbers within circles or overlapping areas indicating the number of OTU's in common to the corresponding diets.

Determination of a core microbiome was accomplished by comparing all solid and liquid samples from all heifers across all diets. Any taxa found to be ubiquitous across all samples were then defined to be part of the core microbiome of the rumen. Similar analysis was performed for each dietary treatment, comparing both the solid and liquid digesta from all samples from all heifers. When solid and liquid samples were combined for each animal, an analysis was done to determine which bacterial taxa were prevalent in the whole rumen contents of all animals on each of the three major dietary regimes (forage, mixed forage and high grain). From these data Venn diagrams were constructed (Figure 4 and 5).

The overall core microbiome was found to consist of the phyla *Bacteroidetes* (32.8%), *Firmicutes* (43.2%) and *Proteobacteria* (14.3%; Table 3). Both of the largest phyla had corresponding classes and orders that were found to be part of the core microbiome at slightly lower abundances (*Bacteroidia*/*Bacteroidales*, *Clostridia/Clostridiales*, Table 3). *Lachnospiraceae* and *Prevotellaceae* at the family level, as well as *Prevotella* spp. at the genus level were also present in all samples. When individual treatments were analyzed for “core taxa”, those animals fed the forage treatment showed a distinctive core microbiome including 14 additional genera and two additional phyla; *Spirochaetes* and *Fibrobacteres* (Table 3). The mixed forage core taxa showed numerically less total numbers of taxa compared to the forage and high grain diets. The mixed forage diet core microbiome had decreased abundances of the taxa *Rikenellaceae*, *Fibrobacteres*, *Erysipelotrichia*, and *Lachnospiraceae* when compared to the forage microbiome. However, those animals on the mixed forage treatment also had increased abundances of *Prevotella*, *Clostridiaceae* and nearly double the abundance of Proteobacteria when compared to the forage treatment (Table 3). The phyla Proteobacteria and its corresponding class/order/family/genus *Gammaproteobacteria*/*Aeromondales*/*Succinivibrionaceae*/*12*–*18* were part of the core taxa of the high grain as was the phyla *Actinobacteria*. The total number of bacterial taxa present in the core microbiome for the acidotic challenge at 4 h and 12 h post-feeding was decreased compared to diets fed previously (Table 3). The core taxa in heifers during the recovery period differed little from those observed during the acidotic challenge, differing only in *Rikenellaceae* and *Gammaproteobacteria*, both of which had been identified in the core taxa of previous diets.

**Table 3:** Percent abundance of taxa to the “Rumen Core Microbiome” and the dietary microbiomes for forage, mixed forage, high grain, acidotic challenge (4 h post-feeding and 12 h post-feeding) and recovery period shown with ± standard error of the mean.

			Dietary Treatments						
		Rumen core	Forage	Mixed forage	High grain		Acidotic challenge		Recovery
Taxa level	Classification					4 h PF		12 h PF	
Phyla	*Actinobacteria*				1.6±0.4			1.4±0.2	
Phyla	*Bacteroidetes*	32.8±1.4	25.7±3.0	26.2±3.6	40.3±2.0	40±2.2		34.5±4.3	31.5±2.6
Class	*Bacteroidia*	31.1±1.4	24.1±2.7	24.9±3.5	37.7±2.4	37.8±2.7		33.6±3.9	29.8±3.0
Order	*Bacteroidales*	31.1±1.4	24.1±2.7	24.9±3.5	37.7±2.4	37.8±2.7		33.6±3.9	29.8±3.0
Family	*S24-7*		3.8±1.7	2.2±0.5					
	*BS11* 1		2.3±0.3						
Family	*Prevotellaceae*	24.7±1.6	12±1.7	16.3±3.1	32.7±2.8	33.2±3.0		29.9±3.9	25.5±3.2
Genus	*Prevotella*	22.2±1.7	8.9±1.0	12.8±2.9	31.6±3.0	30.3±3.1		28.3±3.9	24.1±2.9
Genus	*12-129*		1.6±0.3						
Genus	*BF34*		1.2±0.2						
Family	*Rikenellaceae*		3.7±0.4	3.0±0.5					1.4±0.3
Genus	*RC9*		3.3±0.3	2.7±0.5					1.2±0.3
Phyla	*Cyanobacteria*				1.8±0.6				
Class	*4C0d-2*				1.7±0.6				
Phyla	*Fibrobacteres*		7.1±1.5						
Class	*Fibrobacteria*		7.1±1.5						
Order	*Fibrobacterales*		7.1±1.5						
Family	*Fibrobacteraceae*		7.1±1.5						
Genus	*Fibrobacter*		7.1±1.5						
Phyla	*Firmicutes*	43.2±1.9	55.2±2.4	55.8±4.1	37±0.9	33.6±3.5		37.2±4.5	43.7±3.9
Class	*Erysipelotrichia*		1.8±0.3						
Order	*Erysipelotrichales*		1.8±0.3						
Family	*Erysipelotrichaceae*		1.8±0.3						
Class	*Clostridia*	40.5±2.0	53.3±2.4	53.9±4.4	34.9±0.8	31.1±3.6		32±4.3	41.7±3.8
Order	*Clostridiales*	40.3±2.0	53.1±2.4	53.4±4.5	34.5±0.8	31.0±3.6		31.9±4.3	41.5±3.8
Family	*Ruminococcaceae*		17.3±0.9						
Genus	*Papillibacter*		1.3±0.2						
Genus	*RFN71*		1.6±0.2						
Genus	*Ruminococcus_1*		1.7±0.2						
Genus	*SP3-e02_2*		0.9±0.2						
Genus	*Saccharofermentans*		2.4±0.4						
Genus	*vadinHA42*		4.0±0.2						
Family	*Lachnospiraceae*	19.3±1.4	32.1±2.0	22.7±3.8	16.9±2.1	12.2±2.5		15.0±3.4	18.3±2.5
Genus	*Acetitomaculum*		0.9±0.1						
Genus	*Butyrivibrio_fibrisolvens*		2.3±0.4						
Genus	*RFN8-YE57*		10.9±1.3						
Family	*Clostridiaceae*		0.9±0.1	1.2±0.2					
Phyla	*Proteobacteria*	14.3±1.8	4.7±0.8	8.9±2.3	17.9±2.3	16.5±5.0		20.1±6.9	15.2±3.0
Class	*Gammaproteobacteria*				14.7±2.0	14.8±5.0		17.9±6.4	12.6±3.3
Order	*Aeromonadales*				14.3±2.1	14.5±5.0		17.7±6.4	
Family	*Succinivibrionaceae*				14.3±2.1	14.5±5.0		17.7±6.4	
Genus	*12-18*				7.2±1.5				
Class	*Alphaproteobacteria*		2.3±0.7			1.3±0.3			
Phyla	*Spirochaetes*		2.8±0.7						
Class	*Spirochaetes*		2.8±0.7						
Order	*Spirochaetales*		2.8±0.7						5.0±3.0
Family	*Spirochaetaceae*		2.7±0.8						
Genus	*Treponema*		2.7±0.8						

Averages include all animals, with both liquid and solid digesta values combined within each sampling time point.

^1^ BS11 is not designated to any family within the order Bacteroidales.

### Effects of an acidotic challenge on rumen microbes

**Figure 6:**
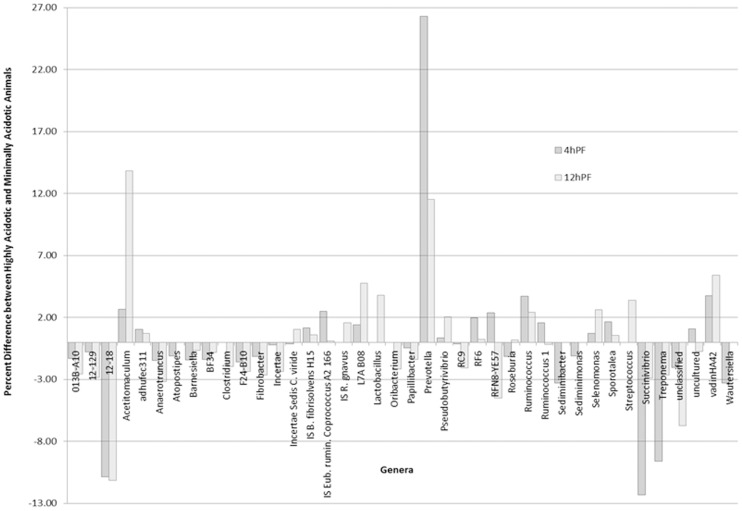
Difference in relative abundance of bacterial populations in clinically vs subclinically acidotic heifers. The graph depicts the difference in relative abundance (%) of genera in clinically acidotic heifers (7 and 41) compared with subclinically acidotic heifers (143 and 153) at 4 h and 12 h post-challenge. Acidotic ranking was determined by area under the curve for pH<5.2 adjusted for animal DMI.

Responses of the individual heifers to the acidotic challenge has been previously reported [19]. Two of the eight heifers (7 and 41) used in the study showed the lowest pH (4.00 and 3.93, respectively) and the highest area under pH 5.2 during the acidotic challenge. These animals were considered to be clinically acidotic, as clear reductions in feed intake were observed and ruminal lactate levels were high. These two heifers were administered sodium bicarbonate at 1630 h as per animal care guidelines; several hours after sampling of ruminal contents for microbiome analysis. In contrast, heifers 143 and 153 responded less severely to the challenge (mean daily pH of 6.14 and 6.25, and the area under pH 5.2 of 7 and 3 pH × min, respectively) and were considered subclinically acidotic as they lacked high levels of ruminal lactate. The relative abundance of all genera in the samples taken during the acidotic challenge (both 4 and 12 h post-feeding samples) were used to describe the impact of acidosis on the bacterial populations in both liquid and solid- rumen contents (Figure 6). Populations were compared between clinically acidotic (7 and 41) and subclinically acidotic (143 and 153) heifers. *Prevotella* exhibited the greatest change between clinical and subclinical acidotic groups with more than a 26% increase 4 h post-acidotic challenge and an 11% increase 12 h post-challenge in the clinically acidotic heifers (Figure 6). *Acetitomaculum*, *L7A B08*, *Pseudobutyrivibrio*, *Selenomonas*, and *vadinHA42* genera also increased at 4 h and at 12 h post-acidotic challenge in clinically acidotic heifers, with the most dramatic increase being in *Acetitomaculum* (11% increase; Figure 6). *Lactobacillus* and *Streptococcus* were the only bacteria that were not present 4 h post-acidotic challenge but were obvious in clinically acidotic heifers 12 h post-challenge (3.7% and 3.4%, respectively). The most dramatic increase in abundant genera level was found in heifers that were subclinically acidotic and consisted of an unidentified rumen bacterium *12*–*18*, as well as *Succinivibrio*, and *Treponema* (10.8%, 12.3% and 9.6% higher at 4 h post-feeding, respectively). Of these, only *12*–*18* further increased between 4 and 12 h post-acidotic challenge. Though a number of genera were found to be higher in subclinically acidotic heifers, most of these constituted <3% change in the bacterial population. *RFN8-YE57* also, increased by 2.4% in clinically acidotic heifers at 4 h post-challenge, but was 4.5% lower than subclinical heifers 12 h after challenge (Figure 6).

A correlation analysis of the key pH variables (minimum pH and pH area under 5.2) to all classified genera was performed and 6 genera in the solid and liquid-digesta samples were correlated to one or more variables (Table 4). *Prevotella* increased (*P*<0.001) in the solid and liquid rumen contents as minimum pH decreased and as pH area less than 5.2 increased. The liquid and solid digesta associated *RFN8-YE57* responded (*P*≤0.05) to pH decreasing in total number as pH decreased or as pH area less than 5.2 increased. Other *genera* that also decreased as daily mean pH decreased included *IS Eub*. *hallii*, and *vadinHA42*. Conversely, *Acidaminococcus*, and *IS Eub*. *rumin Coprococcus A2166I* increased in percent abundance as minimum pH decreased. All *genera* that increased in response to minimum pH, also increased as pH area under 5.2 increased.

**Table 4:** Correlation of key pH parameters to rumen genera in both the solid (S) and liquid (L) digesta of acidotically challenged heifers.

	Correlation				*P*-Value			
	pH minimum		pH area under 5.2 (ph×min)		pH minimum		pH area under 5.2 (ph×min)	
Genus	S	L	S	L	S	L	S	L
*Acidaminococcus*	−0.30	−0.36	0.11	0.19	0.11	0.05	0.56	0.32
*IS Eub. hallii*	0.45	0.31	−0.29	−0.33	0.01	0.09	0.13	0.07
*IS Eub. rumin Coprococcus A2 166*	−0.32	−0.38	0.35	0.48	0.08	0.04	0.06	0.01
*Prevotella*	−0.61	−0.68	0.56	0.61	<0.001	<0.001	<0.001	<0.001
*RFN8-YE57*	0.58	0.60	−0.31	−0.36	<0.001	<0.001	0.10	0.05
*vadinHA42*	0.27	0.38	−0.10	−0.23	0.15	0.04	0.61	0.23

## Discussion

The majority of current knowledge regarding the rumen microbiome has been derived using traditional culturing and enumeration methods[1], [6]. However, many rumen microbes have yet to be isolated/cultured and those which have been represent only a fraction of the total microbiome [2], [35]. Molecular-based methods have helped to further elucidate the dynamics of the rumen ecosystem by predicting evolutionary relationships without the need to cultivate organisms, and can quantify microbes in real time [35], [36], [37]. Furthermore, the use of next generation sequencing of the 16S rRNA gene has revealed the complexity of many gut microbial communities, its variability among animal hosts,and established correlations between imbalances in the gut microbiome and perturbations of host health in humans and cattle [10], [16], [38]. Based on the use of molecular methodology, the aim of this experiment was to characterize the solid and liquid associated bacterial communities in heifers fed forage, mixed forage-grain, and high-grain diets, as well as during an acidotic challenge and after recovery. Additionally, this study aimed to further elucidate a potential ‘core microbiome’ in the rumen, a group of bacterial taxa that can be found in all animals, across a variety of diets, similar to that hypothesized for the human GIT. This study also attempted to determine if individual animal susceptibility to an acidotic challenge was correlated to changes in the rumen microbial ecosystem.

### Impact of Diet

It has also long been understood that diet influences the diversity and community composition of rumen contents [6], [39] and studies using a variety of molecular techniques have been used to elucidate the changes associated with dietary changes and subclinical ruminal acidosis [24], [37], [39], [40]. Similar to these past studies, molecular methods used in this experiment showed the impact of dietary change on the rumen microbial populations of several well known rumen taxa. While qPCR was able to show changes in individual targeted genera or species, DGGE analysis was unable to clearly identify an effect of diet on the overall rumen composition. However, the use of 454 pyrosequencing allowed the authors to classify over 44 distinct genera in this study, which varied significantly based on dietary composition or rumen fraction. Several of these bacteria are among the most commonly researched ruminal bacteria including *Fibrobacter*, *Prevotella*, *Rumminococcus*, *Selenomonas*, *Streptococcus* and *Succinivibrio*. Some of the genera identified in this study have also been found in other culture-dependant and independent studies including *Treponema* [41], [42], [43] and *Ruminobacter* [39]. Furthermore, in this study, a number of genera previously unreported in the rumen were identified as affected by diet, fraction or the interaction of these two factors including *Atopostipes*, *Persicitalea* and *Thalassospira* (Figure 3). While all of these genera have been previously identified as proteobacteria from aquatic environmental samples, only *Atopostipes* has been previously identified in contents from the gastrointestinal tract [44]. Despite the detail determined here from sequencing data, previous estimates have shown that 80,000 sequencing reads should be sufficient to cover all the OTUs in the rumen under any dietary condition [16]. This estimate is supported by the rarefaction curves developed for each dietary treatment in our study. Though 80,000 sequencing reads is potentially excessive for seemingly less diverse diets containing predominantly grain, it is clear that the diversity of forage-based diets (forage and mixed forage) require deeper sequencing in order to obtain full coverage of the rumen microbiome. Jami and Mizrahi [16] were able to sequence up to a maximum of 16,000 sequences per sample in the mature dairy cow whereas Li *et al.* [45] were able to sequence as many as 30,000 reads per sample in the pre-ruminant calf. In the current study, 21,000 to 36,000 sequences were determined for each of the dietary treatments.

Hungate was the first to study alterations in the microflora of the rumen to explain the “microbial actions” causing digestive disturbances in sheep and cattle. He reported that an excess of grain introduced into the rumen caused the cellulolytic bacteria to greatly decrease in numbers while the relative numbers of Gram-positive bacteria increased [46]. This study also presented evidence that *Streptococcus bovis*, a gram-positive organism, was a major contributor to ruminal acidosis. These initial observations on major microbial alterations during ruminal acidosis remain valid; however, there is still a lot unknown about the microbial changes associated with subclinical acidosis [3], [19], [47]. In the current study, *Proteobacteria* increased to as much as 20.1% of the population 12 h after the acidotic challenge, whereas the *Firmicutes*, a gram-positive group, decreased by as much as 10% 4 h post-challenge. This is contradictory to Hungate's proposal that the relative numbers of gram-positive bacteria increase under acidotic conditions [46]. While the details of these changes were unidentifiable at the genus level in this study, these data provide a basis for further research into the core taxa associated with acidosis. Furthermore, Hungate *et al.* [46] stated that numbers of cellulolytic bacteria were greatly decreased as nonvolatile acids accumulated in the rumen, whereas our study shows that over all diets there are minimal cellulolytic bacteria present and therefore decreased numbers of cellulolytic bacteria such as *Ruminococcus* are likely due to dietary changes and not specifically a result of acidosis.

By comparing abundances of bacterial genera affected by diet from heifers that showed a severe response (clinical acidosis) to an acidotic challenge with those that exhibited a lesser response (subclinical acidosis), this study was able to identify a number of critical bacteria associated with clinical acidosis. Similar to the original findings of Hungate *et al.* [46], *Streptococcus* spp. were found to proliferate under acidotic conditions, as did *Lactobacillus* spp. and *Selenomonas* spp. Population increases in a number of other genera as a result of acidosis included *Acetitomaculum*, *L7A-B08*, *Pseudobutyrivibrio* and *vadinHA42*. Of these genera, only *vadinHA42* had not been previously described in the rumen. While *Pseudobutyrivibrio* has been identified as belonging to *Clostridium Cluster* XIVa, the exact metabolic characteristics of this genera and cluster are diverse and therefore warrant further exploration. Also in this study and previously, it has been noted that *Prevotella* and *Succinivibrio* are responsive to perturbations in the rumen environment, increasing in abundance during acidosis [48]. However, due to the ubiquitous presence of *Prevotella* spp. in the rumen as part of the core microbiome, these changes may be simply due to changes in nutrient availability. Analysis of the post challenge recovery period was done to determine the recovery potential of the core microbiome. However, during the recovery period no unique species were identified thus, there was a lack of a clear modification in the core taxa as a result of acidosis (Figure 5). The only notable change in the core taxa as a result of the acidotic challenge was an increase in Rikenella *spp.*, which was previously only found to be associated with the forage diet. The family Rikenellaceae has been previously found in the digestive tracts of cattle [24] fed triticale and is commonly found in the digestive tract of mammals. The metabolic function and role of this family in the rumen microbiome remains to be defined. It is important to note that individual animal variability could potentially have an impact on the results reported here. Since only two animals were used for defining clinical versus subclinical acidosis in this experiment, further research is necessary to determine community differences within a larger group of cattle.

The ability of the rumen microbiome to return to a steady state after a perturbation has been previously documented [49], [50]. Our results show recovery of the rumen microbiome in all heifers within a week after an acidotic challenge, regardless of whether the animal developed clinical or subclinical acidosis. Therefore, similar to the research with piglets, in cattle there may be only a limited window of time shortly after birth where the microbiome is open to significant alterations in its core [51]. Perturbations to the rumen core microbiome outside of this early development window may be of a transient nature with recovery of the microbiome to its initial composition being the most probable outcome [50].

### Impact of Fraction

Rumen bacteria have been classified into three major groups according to their environment, free-living bacteria associated with the liquid digesta, adherent bacteria associated with feed particles and the epimural community which is adherent to the rumen epithelium [5], [52], [53]. Despite the clear delineation between populations, most studies analyze pooled samples of liquid and solid fractions and relatively few molecular studies have examined differences between these populations under various dietary regimes [9], [24], [43], [54]. In our study, results based on the qPCR methodology showed that, of the quantified bacteria, only *F. succinogenes* was significantly higher in solid versus the liquid digesta. With the pyrosequencing methodology only 4 genera showed a significant effect of digesta fraction without any dietary interactions. *Wautersiella*, *IS Eub rumin Coprococcus* and *IS B. fibrisolvens H15* were significantly higher in the solid fraction and *Atopostipes* was only present in the liquid fraction. The predominance of the first three genera in the solid digesta could indicate that these are firmly adherent bacteria and potentially members of the digestive biofilm of the feed surface. Recent pyrosequencing research of the rumen microbiome found a number of bacterial genera were associated with the solid digesta including *B. fibrisolvens* [55], [56]. The presence of *Atopostipes* in only the liquid digesta may indicate that this genus has no role in biofilms and is solely a secondary fermenter that does not need to adhere to particulate matter to acquire nutrients. While supporting data for these genera are unavailable, previous publications have identified *Wautersiella*, *IS Eub rumin Coprococcus* and *IS B. fibrisolvens H15* and *Atopostipes* in various aquatic environments. This implies that these genera may have a similar metabolic role in the liquid rumen environment as in other aqueous environments.

### Core Microbiome

In the past few years, human gastrointestinal microbiology has largely focused on elucidating the ‘core microbiome’, those species that are found in every individual [38], [57], [58] and this concept has also recently been applied in rumen microbial ecology [16], [45]. However, previous studies in ruminants were done using only one dietary regime as the basis of the analysis. One of the largest barriers to determining the core microbial population in humans is the diversity in dietary composition [15]. While each dietary regime can have its own distinct microbial profile, the true ‘core microbiome’ is present regardless of diet composition [59]. In cattle, dietary composition is diverse and based on a number of factors; however, it is easier to control and accurately analyze compared to humans, making the determination of a ‘core microbiome’ for cattle even more feasible. Jami and Mizrahi [16] were able to identify 32 genera across 16 cattle fed a lactation diet whereas Li *et al.* [45] identified 45 genera that were common to 4 calves fed milk-replacer. Unlike this previous research, the current study was only able to identify a single genus, *Prevotella* (22.2%), which was ubiquitous in all 8 heifers across all diets. However, both of the previous studies also found the genus *Prevotella* to be part of the shared microbiome. Classical studies have shown *Prevotella* to be proteolytic and while many species of this genus have the capacity to degrade protein, this genus is present in the rumen across a variety of diets suggesting that this genus exhibits substantial metabolic diversity [28]. When a higher taxa level was used in our study, a more detailed core rumen microbiome was described, as noted in previous studies (Table 3) [16]. The major discrepancy between the findings of the current and previous studies likely arises from the wide range of diets we investigated in our study. Similar to the current study, previous research has also shown that despite swapping of ruminal contents [50] or acidosis [49], the core microbiome is robust and resistant to change. However, the ability of the rumen microbiome to recover within a week of severe acidotic conditions has not been previously documented. The fact that some cattle that experience severe acidosis appear to continue to exhibit erratic feed intake patterns may arise from a permanent physiological rather than a microbial alteration.

## Conclusions

The development and advancement of molecular techniques and their use in complex ecosystems such as the rumen has reinitiated investigations into the basic rumen microbial ecology questions raised more than 50 years ago. These modern methods are confirming and expanding the classical microbiological findings of Hungate and others. Our study demonstrates that the core rumen microbiome is surprisingly stable across a range of diets and during an acidotic event. Alterations in community structure that do occur during acidosis appear to quickly recover after a few days. Bacteria that emerge or undergo significant population changes could serve as indicators of subclinical or clinical acidosis. Further research to determine if there is a possibility for microbial programming or alteration of rumen microbial succession may identify the optimal time to manipulate the core microbiome in a manner that could lead to substantive improvements in ruminant productivity such as resistance to digestive upset or increased feed efficiency.

## Supporting Information

Figure S1 **Rarefaction curves for rumen bacterial communities for each dietary treatment.** Curves depicting the average number of unique OTU's as a fraction of the total number of sequences obtained. Each curve represents a treatment average based on multiple heifers with the solid and the liquid fractions for each treatment combined. Unique OTU's are estimated at a 10% difference level.
(TIF)

Table S1 **Summary of dietary treatment comparisons for unique OTUs, richness estimates, and diversity indices.** The minimum number of unique OTUs in each population was determined with a 10% difference level.
(DOC)

Table S2 **Percent contribution of genus level epithelial taxa to the rumen microbial populations averaged over all treatments for individual animals.** Treatments include forage, mixed forage, high grain, acidotic challenge and challenge recovery. Remaining genera not shown due to non-significant differences between treatments.
(DOC)

Table S3 **Correlation of all calculated pH variables from the acidotic challenge treatment to epithelial genera.** Only those genera found to be significant are shown. acidotic challenge pH variables are the mean for all animals on that dietary treatment.
(DOC)

Table S4 **Percent contribution of phyla level epithelial taxa to the rumen microbial populations averaged over all treatments for individual animals.** Treatments include forage, mixed forage, high grain, acidotic challenge and challenge recovery.
(DOC)

Table S5 **Correlation of pH variables to epithelial phylum.** Only those phyla found to be significant are shown.
(DOC)

Table S6 **Rumen fermentation variables measured in heifers during dietary transition.** Transition treatment diets included forage, mixed forage, high grain, acidotic challenge and challenge recovery*.
(DOC)

Table S7 **Rumen fermentation parameters including pH, volatile fatty acids and lactic acid averaged in individual cattle over diet transition.** Transition treatment diets included forage, mixed forage, high grain, acidotic challenge and challenge recovery.*
(DOC)
