# Supplementary figures and images for: Characterization of the Core Rumen Microbiome in Cattle during Transition from Forage to Concentrate as Well as during and after an Acidotic Challenge
Source: PLoS One. 2013 Dec 31;8(12):e83424. doi: 10.1371/journal.pone.0083424 (PMC3877040; doi:10.1371/journal.pone.0083424)

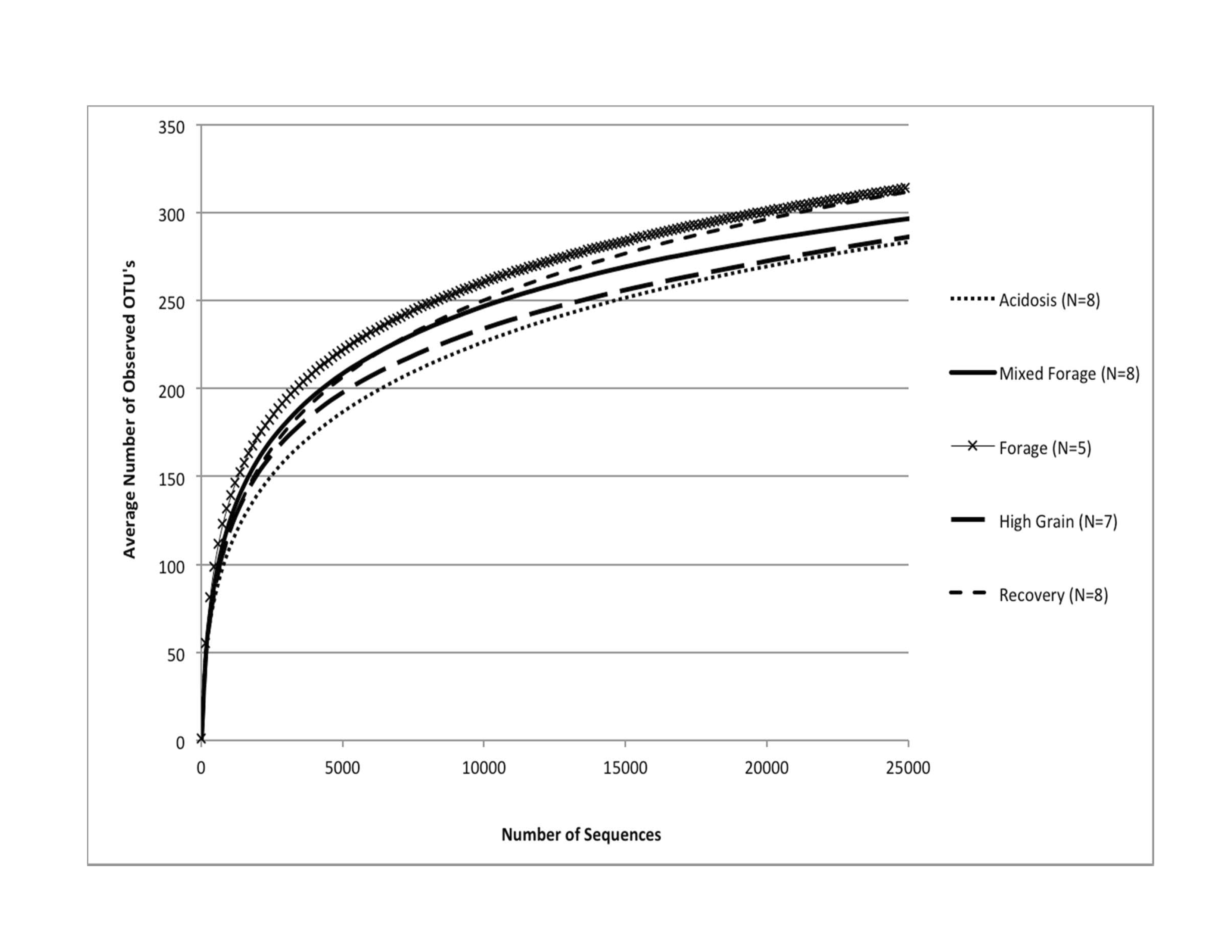

Supplement: Figure S1 — Rarefaction curves for rumen bacterial communities for each dietary treatment. Curves depicting the average number of unique OTU's as a fraction of the total number of sequences obtained. Each curve represents a treatment average based on multiple heifers with the solid and the liquid fractions for each treatment combined. Unique OTU's are estimated at a 10% difference level. (TIF) [file pone.0083424.s001.tif]
