# Supplementary material for: Characterization of the Core Rumen Microbiome in Cattle during Transition from Forage to Concentrate as Well as during and after an Acidotic Challenge
Source: PLoS One. 2013 Dec 31;8(12):e83424. doi: 10.1371/journal.pone.0083424 (PMC3877040; doi:10.1371/journal.pone.0083424)
Supplement: Table S1 — Summary of dietary treatment comparisons for unique OTUs, richness estimates, and diversity indices. The minimum number of unique OTUs in each population was determined with a 10% difference level. (DOC) [file pone.0083424.s002.doc]

**TABLE S1. Summary of dietary treatment comparisons for unique OTUs, richness estimates, and diversity indices. The minimum number of unique OTUs in each population was determined with a 10 % difference level.**

|  | **Forage** | **Mixed forage** | **High grain** | **Acidotic challenge** | **Challenge recovery** | **SEM** | ***P*-value** |
| --- | --- | --- | --- | --- | --- | --- | --- |
| Number of sequences | 5045 | 3731 | 4349 | 4148 | 4389 | 579 | 0.68 |
| Coverage (%) | 99.0 | 98.8 | 99.1 | 98.9 | 99.0 | 0.2 | 0.64 |
| Total # of unique OTUs | 161 | 147 | 144 | 148 | 149 | 11 | 0.90 |
| Richness estimate |  |  |  |  |  |  |  |
| Chao1 | 198 | 181 | 183 | 192 | 181 | 16 | 0.94 |
| ACE | 192 | 180 | 179 | 192 | 189 | 15 | 0.94 |
| Diversity indices |  |  |  |  |  |  |  |
| Shannon-Weiner | 3.72 | 3.36 | 3.31 | 3.43 | 3.19 | 0.14 | 0.12 |
| Simpson's | 0.95 | 0.92 | 0.91 | 0.93 | 0.89 | 0.02 | 0.16 |
