# Supplementary material for: Characterization of the Core Rumen Microbiome in Cattle during Transition from Forage to Concentrate as Well as during and after an Acidotic Challenge
Source: PLoS One. 2013 Dec 31;8(12):e83424. doi: 10.1371/journal.pone.0083424 (PMC3877040; doi:10.1371/journal.pone.0083424)
Supplement: Table S2 — Percent contribution of genus level epithelial taxa to the rumen microbial populations averaged over all treatments for individual animals. Treatments include forage, mixed forage, high grain, acidotic challenge and challenge recovery. Remaining genera not shown due to non-significant differences between treatments. (DOC) [file pone.0083424.s003.doc]

**TABLE S2. Percent contribution of genus level epithelial taxa to the rumen microbial populations averaged over all treatments for individual animals. Treatments include forage, mixed forage, high grain, acidotic challenge and challenge recovery. Remaining genera not shown due to non-significant differences between treatments.**

|  | **Individual animals** | | | | | | | |  |  |
| --- | --- | --- | --- | --- | --- | --- | --- | --- | --- | --- |
| **Genera** | **7** | **41** | **43** | **143** | **153** | **156** | **315** | **346** | **SEM** | ***P-*Value** |
| *12-18* | 0.0 | 0.0 | 0.0 | 0.0 | 1.6 | 2.5 | 8.1 | 1.2 | 0.7 | 0.07 |
| *adhufec405* | 2.6 | 0.0 | 1.1 | 0.0 | 0.0 | 2.3 | 8.1 | 4.5 | 0.8 | 0.11 |
| *Atopobacter* | 0.0 | 0.0 | 0.0 | 0.0 | 0.0 | 0.0 | 0.0 | 2.3 | 0.2 | 0.06 |
| *Carboxydibrachium* | 0.0 | 1.1 | 0.0 | 2.6 | 6.3 | 0.0 | 0.0 | 1.4 | 0.6 | 0.11 |
| *Comamonas* | 15.9a | 5.6a | 38.1b | 40.8b | 29.8b | 34.2b | 35.4b | 29.4b | 3.1 | 0.04 |
| *Dialister* | 0.0 | 1.3 | 1.3 | 0.0 | 1.4 | 3.5 | 10.6 | 3.2 | 0.9 | 0.07 |
| *F24-B10* | 0.0a | 0.0a | 0.0a | 0.0a | 0.0a | 0.0a | 5.4b | 0.0a | 0.3 | <0.001 |
| *Guggenheimella* | 4.3ab | 2.7a | 6.8ab | 10.9b | 5.7ab | 11.8b | 2.8ab | 9.4ab | 0.8 | 0.03 |
| *IS Eub. cellulosolvens* | 5.3 | 2.1 | 0.0 | 0.0 | 1.3 | 0.0 | 0.0 | 1.4 | 0.5 | 0.09 |
| *Oxobacter* | 11.4 | 5.7 | 11.3 | 5.2 | 1.4 | 2.8 | 0.0 | 9.6 | 1.2 | 0.06 |
| *p-4496-6Wb3* | 2.5ab | 0.0a | 1.6ab | 9.7b | 2.2ab | 0.0a | 5.7ab | 2.5ab | 0.7 | 0.02 |
| *Pelospora* | 15.4ab | 15.2ab | 15.8ab | 31.6b | 3.2a | 21.2ab | 11.1ab | 10.4ab | 2.0 | 0.03 |
| *rc1-13* | 4.2 | 2.9 | 5.2 | 13.4 | 8.5 | 4.3 | 12.5 | 3.9 | 1.1 | 0.07 |
| *RC20* | 0.0a | 0.0a | 0.0a | 0.0a | 7.0b | 0.0a | 0.0a | 1.4ab | 0.6 | 0.01 |
| *Succinivibrio* | 0.0 | 0.0 | 1.3 | 0.0 | 0.0 | 3.1 | 9.2 | 3.9 | 0.8 | 0.07 |
| *Thermotalea* | 0.0a | 4.9b | 4.1b | 4.0b | 4.2b | 0.0a | 0.0a | 0.0a | 0.6 | 0.05 |
| *U29-B03* | 0.0a | 0.0a | 0.0a | 0.0a | 0.0a | 3.5b | 0.0a | 0.0a | 0.2 | <0.001 |
| *uncultured* | 9.4ab | 8.6ab | 13.3b | 4.2a | 16.5b | 4.3a | 2.7a | 10.9ab | 1.2 | 0.05 |
| *Verminephrobacter* | 0.0a | 0.0a | 0.0a | 6.4b | 1.9ab | 0.0a | 1.8ab | 4.2b | 0.6 | 0.03 |
| *wet75* | 9.1ab | 3.1a | 7.5ab | 11.4ab | 11.8ab | 19.01b | 2.5a | 7.8ab | 1.2 | 0.05 |
