# Supplementary material for: Characterization of the Core Rumen Microbiome in Cattle during Transition from Forage to Concentrate as Well as during and after an Acidotic Challenge
Source: PLoS One. 2013 Dec 31;8(12):e83424. doi: 10.1371/journal.pone.0083424 (PMC3877040; doi:10.1371/journal.pone.0083424)
Supplement: Table S3 — Correlation of all calculated pH variables from the acidotic challenge treatment to epithelial genera. Only those genera found to be significant are shown. acidotic challenge pH variables are the mean for all animals on that dietary treatment. (DOC) [file pone.0083424.s004.doc]

**TABLE S3**. Correlation of all calculated pH variables from the acidotic challenge treatment to epithelial genera. Only those genera found to be significant are shown. acidotic challenge pH variables are the mean for all animals on that dietary treatment.

|  |  | **pH variables** | | | | | | | | |
| --- | --- | --- | --- | --- | --- | --- | --- | --- | --- | --- |
| **Genera** |  | **pH min** | **pH mean** | **pH max** | **Duration under pH 5.8 (min)** | **pH area under 5.8 (pH×min)** | **Duration under pH 5.5 (min)** | **pH area under 5.5 (pH×min)** | **Duration under pH 5.2 (min)** | **pH area under 5.2 (pH×min)** |
| *Acetitomaculum* | Correlation | -0.32 | -0.31 | 0.19 | 0.20 | 0.45 | 0.24 | 0.52 | 0.24 | 0.57 |
|  | *P*-Value | 0.09 | 0.10 | 0.32 | 0.29 | 0.01 | 0.20 | 0.00 | 0.22 | 0.00 |
| *Acidaminococcus* | Correlation | -0.32 | -0.33 | 0.16 | 0.27 | 0.39 | 0.26 | 0.42 | 0.24 | 0.42 |
|  | *P*-Value | 0.09 | 0.08 | 0.41 | 0.16 | 0.04 | 0.17 | 0.03 | 0.21 | 0.02 |
| *Anaerophaga* | Correlation | -0.38 | -0.61 | 0.12 | 0.25 | 0.62 | 0.32 | 0.71 | 0.41 | 0.80 |
|  | *P*-Value | 0.04 | 0.00 | 0.52 | 0.18 | 0.00 | 0.09 | 0.00 | 0.03 | 0.00 |
| *Anaerovorax* | Correlation | 0.34 | 0.44 | 0.12 | -0.43 | -0.40 | -0.45 | -0.36 | -0.38 | -0.31 |
|  | *P*-Value | 0.07 | 0.02 | 0.53 | 0.02 | 0.03 | 0.02 | 0.05 | 0.04 | 0.11 |
| *Atopobacter* | Correlation | -0.30 | -0.17 | 0.16 | 0.38 | 0.30 | 0.36 | 0.25 | 0.24 | 0.14 |
|  | *P*-Value | 0.11 | 0.39 | 0.41 | 0.04 | 0.11 | 0.06 | 0.19 | 0.20 | 0.47 |
| *Atopobium* | Correlation | -0.61 | -0.58 | -0.37 | 0.76 | 0.61 | 0.73 | 0.51 | 0.60 | 0.35 |
|  | *P*-Value | 0.00 | 0.00 | 0.05 | 0.00 | 0.00 | 0.00 | 0.00 | 0.00 | 0.06 |
| *Azonexus* | Correlation | 0.39 | 0.40 | -0.06 | -0.50 | -0.39 | -0.48 | -0.32 | -0.43 | -0.25 |
|  | *P*-Value | 0.04 | 0.03 | 0.75 | 0.01 | 0.04 | 0.01 | 0.09 | 0.02 | 0.18 |
| *Bacteroides* | Correlation | -0.11 | -0.34 | -0.07 | 0.07 | 0.29 | 0.13 | 0.35 | 0.20 | 0.39 |
|  | *P*-Value | 0.57 | 0.08 | 0.72 | 0.72 | 0.13 | 0.52 | 0.06 | 0.30 | 0.03 |
| *Butyrivibrio fibrisolvens* | Correlation | 0.55 | 0.49 | 0.20 | -0.59 | -0.52 | -0.57 | -0.46 | -0.51 | -0.38 |
|  | *P*-Value | 0.00 | 0.01 | 0.31 | 0.00 | 0.00 | 0.00 | 0.01 | 0.00 | 0.04 |
| *Carboxydibrachium* | Correlation | 0.40 | 0.27 | 0.26 | -0.34 | -0.27 | -0.34 | -0.23 | -0.30 | -0.16 |
|  | *P*-Value | 0.03 | 0.15 | 0.18 | 0.07 | 0.15 | 0.07 | 0.23 | 0.11 | 0.40 |
| *cc142* | Correlation | -0.43 | -0.26 | -0.17 | 0.43 | 0.24 | 0.38 | 0.15 | 0.31 | 0.03 |
|  | *P*-Value | 0.02 | 0.18 | 0.38 | 0.02 | 0.21 | 0.04 | 0.43 | 0.11 | 0.87 |
| *Comamonas* | Correlation | 0.17 | 0.17 | -0.06 | -0.07 | -0.30 | -0.20 | -0.36 | -0.27 | -0.39 |
|  | *P*-Value | 0.38 | 0.37 | 0.78 | 0.71 | 0.11 | 0.29 | 0.06 | 0.16 | 0.04 |
| *Dialister* | Correlation | -0.32 | -0.36 | 0.06 | 0.37 | 0.34 | 0.36 | 0.31 | 0.39 | 0.28 |
|  | *P*-Value | 0.09 | 0.06 | 0.74 | 0.05 | 0.07 | 0.05 | 0.11 | 0.03 | 0.13 |
| *Eub. brachy* | Correlation | 0.48 | 0.35 | 0.00 | -0.37 | -0.26 | -0.31 | -0.22 | -0.28 | -0.17 |
|  | *P*-Value | 0.01 | 0.06 | 0.99 | 0.05 | 0.17 | 0.10 | 0.26 | 0.14 | 0.37 |
| *Fervidicola* | Correlation | 0.43 | 0.34 | -0.06 | -0.33 | -0.28 | -0.34 | -0.24 | -0.35 | -0.18 |
|  | *P*-Value | 0.02 | 0.07 | 0.75 | 0.08 | 0.15 | 0.08 | 0.22 | 0.06 | 0.35 |
| *Fusobacterium* | Correlation | 0.48 | 0.37 | 0.61 | -0.31 | -0.25 | -0.27 | -0.22 | -0.21 | -0.18 |
|  | *P*-Value | 0.01 | 0.05 | 0.00 | 0.10 | 0.19 | 0.16 | 0.26 | 0.28 | 0.36 |
| *Incertae Sedis C. viride* | Correlation | 0.47 | 0.33 | -0.06 | -0.35 | -0.24 | -0.29 | -0.20 | -0.27 | -0.16 |
|  | *P*-Value | 0.01 | 0.08 | 0.76 | 0.06 | 0.20 | 0.12 | 0.31 | 0.15 | 0.42 |
| *IS B. fibrisolvens H15* | Correlation | -0.36 | -0.54 | 0.11 | 0.34 | 0.56 | 0.36 | 0.61 | 0.47 | 0.66 |
|  | *P*-Value | 0.05 | 0.00 | 0.55 | 0.07 | 0.00 | 0.05 | 0.00 | 0.01 | 0.00 |
| *IS cTPY-17 adhufec52* | Correlation | -0.49 | -0.45 | -0.04 | 0.33 | 0.47 | 0.40 | 0.48 | 0.55 | 0.54 |
|  | *P*-Value | 0.01 | 0.01 | 0.84 | 0.08 | 0.01 | 0.03 | 0.01 | 0.00 | 0.00 |
| *IS R. gnavus* | Correlation | -0.38 | -0.61 | 0.12 | 0.25 | 0.62 | 0.32 | 0.71 | 0.41 | 0.80 |
|  | *P*-Value | 0.04 | 0.00 | 0.52 | 0.18 | 0.00 | 0.09 | 0.00 | 0.03 | 0.00 |
| *Lactobacillus* | Correlation | -0.57 | -0.66 | 0.09 | 0.36 | 0.74 | 0.43 | 0.84 | 0.53 | 0.93 |
|  | *P*-Value | 0.00 | 0.00 | 0.64 | 0.05 | 0.00 | 0.02 | 0.00 | 0.00 | 0.00 |
| *Marvinbryantia* | Correlation | 0.45 | 0.42 | -0.15 | -0.45 | -0.40 | -0.45 | -0.36 | -0.41 | -0.30 |
|  | *P*-Value | 0.01 | 0.02 | 0.44 | 0.01 | 0.03 | 0.01 | 0.06 | 0.03 | 0.12 |
| *Megasphaera* | Correlation | -0.52 | -0.63 | 0.09 | 0.33 | 0.72 | 0.40 | 0.82 | 0.48 | 0.90 |
|  | *P*-Value | 0.00 | 0.00 | 0.64 | 0.08 | 0.00 | 0.03 | 0.00 | 0.01 | 0.00 |
| *Mitsuokella* | Correlation | -0.38 | -0.61 | 0.12 | 0.25 | 0.62 | 0.32 | 0.71 | 0.41 | 0.80 |
|  | *P*-Value | 0.04 | 0.00 | 0.52 | 0.18 | 0.00 | 0.09 | 0.00 | 0.03 | 0.00 |
| *Olsenella* | Correlation | -0.61 | -0.51 | -0.05 | 0.41 | 0.58 | 0.45 | 0.61 | 0.57 | 0.67 |
|  | *P*-Value | 0.00 | 0.00 | 0.79 | 0.03 | 0.00 | 0.01 | 0.00 | 0.00 | 0.00 |
| *rc1-13* | Correlation | 0.49 | 0.50 | -0.09 | -0.50 | -0.52 | -0.54 | -0.49 | -0.51 | -0.42 |
|  | *P*-Value | 0.01 | 0.01 | 0.63 | 0.01 | 0.00 | 0.00 | 0.01 | 0.01 | 0.02 |
| *RC25* | Correlation | 0.35 | 0.30 | -0.29 | -0.39 | -0.32 | -0.39 | -0.26 | -0.36 | -0.21 |
|  | *P*-Value | 0.06 | 0.11 | 0.13 | 0.03 | 0.09 | 0.04 | 0.17 | 0.05 | 0.28 |
| *RC39* | Correlation | -0.75 | -0.71 | -0.08 | 0.64 | 0.79 | 0.69 | 0.79 | 0.70 | 0.77 |
|  | *P*-Value | 0.00 | 0.00 | 0.68 | 0.00 | 0.00 | 0.00 | 0.00 | 0.00 | 0.00 |
| *RF21* | Correlation | 0.44 | 0.45 | -0.02 | -0.35 | -0.35 | -0.31 | -0.34 | -0.29 | -0.34 |
|  | *P*-Value | 0.02 | 0.01 | 0.91 | 0.07 | 0.06 | 0.10 | 0.07 | 0.13 | 0.07 |
| *RF38* | Correlation | 0.52 | 0.38 | 0.08 | -0.43 | -0.35 | -0.41 | -0.30 | -0.35 | -0.24 |
|  | *P*-Value | 0.00 | 0.04 | 0.67 | 0.02 | 0.06 | 0.03 | 0.11 | 0.06 | 0.20 |
| *RFN71* | Correlation | 0.30 | 0.35 | -0.36 | -0.36 | -0.42 | -0.44 | -0.40 | -0.50 | -0.35 |
|  | *P*-Value | 0.12 | 0.06 | 0.06 | 0.05 | 0.02 | 0.02 | 0.03 | 0.01 | 0.07 |
| *RFN8-YE57* | Correlation | 0.42 | 0.42 | 0.06 | -0.41 | -0.42 | -0.42 | -0.40 | -0.38 | -0.32 |
|  | *P*-Value | 0.02 | 0.02 | 0.77 | 0.03 | 0.02 | 0.02 | 0.03 | 0.04 | 0.09 |
| *Ruminococcus 1* | Correlation | 0.43 | 0.46 | 0.07 | -0.42 | -0.40 | -0.42 | -0.37 | -0.42 | -0.32 |
|  | *P*-Value | 0.02 | 0.01 | 0.73 | 0.02 | 0.03 | 0.02 | 0.05 | 0.02 | 0.09 |
| *Saccharofermentans* | Correlation | 0.61 | 0.52 | -0.04 | -0.59 | -0.51 | -0.58 | -0.44 | -0.50 | -0.36 |
|  | *P*-Value | 0.00 | 0.00 | 0.86 | 0.00 | 0.01 | 0.00 | 0.02 | 0.01 | 0.05 |
| *Selenomonas* | Correlation | -0.38 | -0.61 | 0.12 | 0.25 | 0.62 | 0.32 | 0.71 | 0.41 | 0.80 |
|  | *P*-Value | 0.04 | 0.00 | 0.52 | 0.18 | 0.00 | 0.09 | 0.00 | 0.03 | 0.00 |
| *Solobacterium* | Correlation | -0.63 | -0.59 | -0.01 | 0.55 | 0.57 | 0.60 | 0.52 | 0.62 | 0.46 |
|  | *P*-Value | 0.00 | 0.00 | 0.97 | 0.00 | 0.00 | 0.00 | 0.00 | 0.00 | 0.01 |
| *Streptococcus* | Correlation | -0.43 | -0.63 | 0.12 | 0.28 | 0.66 | 0.35 | 0.76 | 0.44 | 0.85 |
|  | *P*-Value | 0.02 | 0.00 | 0.55 | 0.14 | 0.00 | 0.06 | 0.00 | 0.02 | 0.00 |
| *Succiniclasticum* | Correlation | -0.43 | -0.42 | -0.29 | 0.60 | 0.47 | 0.57 | 0.39 | 0.42 | 0.27 |
|  | *P*-Value | 0.02 | 0.02 | 0.12 | 0.00 | 0.01 | 0.00 | 0.04 | 0.02 | 0.16 |
| *Succinivibrio* | Correlation | -0.41 | -0.43 | -0.07 | 0.49 | 0.39 | 0.53 | 0.32 | 0.61 | 0.23 |
|  | *P*-Value | 0.03 | 0.02 | 0.74 | 0.01 | 0.04 | 0.00 | 0.10 | 0.00 | 0.23 |
| *Thermodesulfobium* | Correlation | 0.58 | 0.49 | 0.44 | -0.51 | -0.48 | -0.52 | -0.43 | -0.49 | -0.36 |
|  | *P*-Value | 0.00 | 0.01 | 0.02 | 0.00 | 0.01 | 0.00 | 0.02 | 0.01 | 0.05 |
| *uncultured* | Correlation | 0.56 | 0.60 | 0.04 | -0.48 | -0.53 | -0.49 | -0.52 | -0.52 | -0.51 |
|  | *P*-Value | 0.00 | 0.00 | 0.83 | 0.01 | 0.00 | 0.01 | 0.00 | 0.00 | 0.00 |
| *vadinHA42* | Correlation | 0.40 | 0.37 | 0.08 | -0.28 | -0.35 | -0.32 | -0.35 | -0.34 | -0.31 |
|  | *P*-Value | 0.03 | 0.05 | 0.69 | 0.14 | 0.06 | 0.10 | 0.06 | 0.08 | 0.10 |
| *wet75* | Correlation | 0.22 | 0.20 | -0.17 | -0.05 | -0.32 | -0.22 | -0.38 | -0.38 | -0.41 |
|  | *P*-Value | 0.25 | 0.30 | 0.37 | 0.81 | 0.09 | 0.26 | 0.04 | 0.04 | 0.03 |
