# Supplementary material for: Characterization of the Core Rumen Microbiome in Cattle during Transition from Forage to Concentrate as Well as during and after an Acidotic Challenge
Source: PLoS One. 2013 Dec 31;8(12):e83424. doi: 10.1371/journal.pone.0083424 (PMC3877040; doi:10.1371/journal.pone.0083424)
Supplement: Table S4 — Percent contribution of phyla level epithelial taxa to the rumen microbial populations averaged over all treatments for individual animals. Treatments include forage, mixed forage, high grain, acidotic challenge and challenge recovery. (DOC) [file pone.0083424.s005.doc]

**TABLE S4**. Percent contribution of phyla level epithelial taxa to the rumen microbial populations averaged over all treatments for individual animals. Treatments include forage, mixed forage, high grain, acidotic challenge and challenge recovery.

|  | **Treatment** | | | | |  |  |
| --- | --- | --- | --- | --- | --- | --- | --- |
| **Phyla** | **forage** | **mixed forage** | **high grain** | **acidotic challenge** | **Challenge**  **Recovery** | **SEM** | ***P-*Value** |
| *Actinobacteria* | 2.05a | 1.89a | 3.39ab | 4.93b | 2.60a | 0.51 | 0.0001 |
| *Bacteroidetes* | 9.86 | 7.87 | 9.05 | 8.42 | 12.81 | 1.60 | 0.34 |
| *Candidate divison TM7* | 1.58c | 1.30bc | 0.26a | 0.46ab | 0.28a | 0.12 | 0.0003 |
| *Chloroflexi* | 0.00 | 0.00 | 0.00 | 0.09 | 0.00 | 0.00 | 0.49 |
| *Fibrobacteres* | 0.44 | 0.11 | 0.16 | 0.16 | 0.07 | 0.03 | 0.63 |
| *Firmicutes* | 66.88 | 73.65 | 74.67 | 74.56 | 72.03 | 12.12 | 0.11 |
| *Fusobacteria* | 2.58b | 0.11a | 0.10a | 0.00a | 0.00a | 0.07 | 0.01 |
| *Planctomycetes* | 0.30 | 0.31 | 0.00 | 0.00 | 0.00 | 0.02 | 0.36 |
| *Proteobacteria** | 13.36 | 12.28 | 9.99 | 9.91 | 9.83 | 1.82 | 0.17 |
| *Spirochaetes* | 0.59 | 0.74 | 0.50 | 0.06 | 0.52 | 0.08 | 0.37 |
| *Synergistetes* | 0.17ab | 0.10ab | 0.63b | 0.08a | 0.25ab | 0.04 | 0.05 |
| *Tenericutes* | 0.92b | 0.38ab | 0.11ab | 0.00a | 0.14ab | 0.04 | 0.05 |
|  |  |  |  |  |  |  |  |

*Significant animal effect noted for *Proteobacteria* (*P*=0.04)

Letters in each row indicate significant difference between treatments.
