# Supplementary material for: Characterization of the Core Rumen Microbiome in Cattle during Transition from Forage to Concentrate as Well as during and after an Acidotic Challenge
Source: PLoS One. 2013 Dec 31;8(12):e83424. doi: 10.1371/journal.pone.0083424 (PMC3877040; doi:10.1371/journal.pone.0083424)
Supplement: Table S5 — Correlation of pH variables to epithelial phylum. Only those phyla found to be significant are shown. (DOC) [file pone.0083424.s006.doc]

**TABLE S5**. Correlation of pH variables to epithelial phylum. Only those phyla found to be significant are shown

|  |  | **pH parameters** | | | | | | | | |
| --- | --- | --- | --- | --- | --- | --- | --- | --- | --- | --- |
| **Phyla** |  | **pH min** | **pH mean** | **pH max** | **Duration under pH 5.8 (min)** | **pH area under 5.8 (pH×min)** | **Duration under pH 5.5 (min)** | **pH area under 5.5 (pH×min)** | **Duration under pH 5.2 (min)** | **pH area under 5.2 (pH×min)** |
| *Actinobacteria* | Correlation | -0.75 | -0.60 | -0.29 | 0.70 | 0.68 | 0.72 | 0.63 | 0.68 | 0.54 |
|  | *P*-value | <0.001 | 0.00 | 0.13 | <0.001 | <0.001 | <0.001 | <0.001 | <0.001 | 0.00 |
| *Candidate division TM7* | Correlation | 0.53 | 0.57 | -0.09 | -0.54 | -0.60 | -0.60 | -0.57 | -0.56 | -0.50 |
|  | *P*-value | 0.00 | 0.00 | 0.65 | 0.00 | 0.00 | 0.00 | 0.00 | 0.00 | 0.01 |
| *Fusobacteria* | Correlation | 0.34 | 0.38 | 0.59 | -0.25 | -0.20 | -0.17 | -0.18 | -0.15 | -0.17 |
|  | *P*-value | 0.07 | 0.04 | 0.00 | 0.19 | 0.30 | 0.39 | 0.34 | 0.44 | 0.38 |
| *Planctomycetes* | Correlation | 0.47 | 0.33 | -0.06 | -0.35 | -0.25 | -0.30 | -0.20 | -0.27 | -0.16 |
|  | *P*-value | 0.01 | 0.08 | 0.77 | 0.06 | 0.20 | 0.12 | 0.30 | 0.15 | 0.41 |
| *Tenericutes* | Correlation | 0.39 | 0.32 | -0.01 | -0.31 | -0.30 | -0.33 | -0.28 | -0.32 | -0.23 |
|  | *P*-value | 0.03 | 0.09 | 0.96 | 0.11 | 0.11 | 0.08 | 0.14 | 0.09 | 0.22 |
