# Supplementary material for: Characterization of the Core Rumen Microbiome in Cattle during Transition from Forage to Concentrate as Well as during and after an Acidotic Challenge
Source: PLoS One. 2013 Dec 31;8(12):e83424. doi: 10.1371/journal.pone.0083424 (PMC3877040; doi:10.1371/journal.pone.0083424)
Supplement: Table S6 — Rumen fermentation variables measured in heifers during dietary transition. Transition treatment diets included forage, mixed forage, high grain, acidotic challenge and challenge recovery*. (DOC) [file pone.0083424.s007.doc]

**TABLE S6.** Rumen fermentation variables measured in heifers during dietary transition. Transition treatment diets included forage, mixed forage, high grain, acidotic challenge and challenge recovery*.

|  | **Treatment** | | | | |  |  |
| --- | --- | --- | --- | --- | --- | --- | --- |
| **Rumen Fermentation Variable** | **Forage**** | **Mixed forage** | **High grain** | **Acidotic challenge** | **Challenge recovery** | **SEM** | ***P*-value** |
| Mean nadir |  | 5.78b | 4.96a | 4.61a | 5.08a | 0.12 | <0.001 |
| Mean daily pH |  | 6.36b | 5.99b | 5.47a | 6.09b | 0.12 | <0.001 |
| Mean maximum pH |  | 6.95b | 6.62a | 7.00b | 6.56a | 0.07 | <0.001 |
| Rumen pH≤5.8 |  |  |  |  |  |  |  |
| Duration (min day^-1^) |  | 115c | 828ab | 1023a | 512b | 103 | <0.001 |
| Area under (pH × min) |  | 25c | 448ab | 833a | 186bc | 106 | <0.001 |
| Rumen pH≤5.5 |  |  |  |  |  |  |  |
| Duration (min day^-1^) |  | 25b | 616a | 879a | 232b | 88 | <0.001 |
| Area under (pH × min) |  | 4b | 229ab | 550a | 77b | 85 | <0.001 |
| Rumen pH≤5.2 |  |  |  |  |  |  |  |
| Duration (min day^-1^) |  | 0c | 414ab | 715a | 101bc | 99 | <0.001 |
| Area under (pH × min) |  | 0b | 78ab | 338a | 29b | 68 | <0.001 |
|  |  |  |  |  |  |  |  |
| Total VFA, m*M* | 76.8a | 91.8ab | 144.2c | 128.1abc | 129.5bc | 5.8 | 0.002 |
| Acetate (A), mmol/100mol | 67.8b | 61.8b | 46.6a | 51.5a | 50.6a | 1.4 | <0.001 |
| Propionate (P), mmol/100mol | 17.8a | 19.7a | 37.5b | 32.3b | 33.1b | 1.5 | <0.001 |
| Butyrate, mmol/100mol | 3.84b | 3.27b | 1.27a | 1.69a | 1.84a | 0.17 | <0.001 |
| Lactic acid, m*M ****** |  |  | 0.71a | 2.96b | 0.03a | 0.41 | 0.02 |

*Letters in each row indicate significant difference between treatments. The pH variables are a mean values for all animals within a dietary treatment for the 24 h period starting at 08:00 h on the day of bacterial sample collection. The VFA and lactic acid concentrations are mean values for all animals on a dietary treatment for samples taken 4 h post-challenge.

** pH values were unavailable during the forage treatment

*** Lactic acid values were unavailable for forage and mixed forage treatment.
