# Supplementary material for: Characterization of the Core Rumen Microbiome in Cattle during Transition from Forage to Concentrate as Well as during and after an Acidotic Challenge
Source: PLoS One. 2013 Dec 31;8(12):e83424. doi: 10.1371/journal.pone.0083424 (PMC3877040; doi:10.1371/journal.pone.0083424)
Supplement: Table S7 — Rumen fermentation parameters including pH, volatile fatty acids and lactic acid averaged in individual cattle over diet transition. Transition treatment diets included forage, mixed forage, high grain, acidotic challenge and challenge recovery.* (DOC) [file pone.0083424.s008.doc]

**TABLE S7.** Rumen fermentation parameters including pH, volatile fatty acids and lactic acid averaged in individual cattle over diet transition. Transition treatment diets included forage, mixed forage, high grain, acidotic challenge and challenge recovery.*

|  | **Individual Animal** | | | | | | | |  |  |
| --- | --- | --- | --- | --- | --- | --- | --- | --- | --- | --- |
| **Fermentation variables** | **7** | **41** | **43** | **143** | **153** | **156** | **315** | **346** | **SEM** | ***P*-value** |
| Mean nadir | 4.79 | 5.23 | 4.90 | 5.21 | 5.46 | 5.08 | 5.09 | 5.08 | 0.10 | 0.26 |
| Mean daily pH | 5.84 | 5.88 | 5.65 | 6.14 | 6.25 | 5.98 | 5.96 | 6.13 | 0.08 | 0.31 |
|  |  |  |  |  |  |  |  |  |  |  |
| Rumen pH≤5.8 |  |  |  |  |  |  |  |  |  |  |
| Duration (min day^-1^) | 788 | 494 | 1002 | 427 | 480 | 651 | 535 | 581 | 78 | 0.06 |
| Area under (pH x min) | 509 | 557 | 625 | 131 | 167 | 319 | 319 | 356 | 77 | 0.27 |
| Rumen pH≤5.5 |  |  |  |  |  |  |  |  |  |  |
| Duration (min day^-1^) | 554 | 455 | 787 | 195 | 272 | 408 | 391 | 446 | 75 | 0.05 |
| Area under (pH x min) | 311 | 415 | 348 | 43 | 53 | 164 | 180 | 206 | 57 | 0.38 |
| Rumen pH≤5.2 |  |  |  |  |  |  |  |  |  |  |
| Duration (min day^-1^) | 342 | 372 | 577 | 57 | 65 | 274 | 454 | 320 | 69 | 0.09 |
| Area under (pH x min) | 178 | 290 | 141 | 7 | 3 | 61 | 122 | 88 | 41 | 0.51 |
|  |  |  |  |  |  |  |  |  |  |  |
| Total VFA, m*M* | 127.2 | 119.8 | 121.2 | 136.2 | 107.6 | 136.5 | 137.1 | 107.9 | 5.8 | 0.43 |
| Acetate (A), mmol/100mol | 50.4a | 57.1b | 53.9ab | 50.8a | 53.9ab | 51.2ab | 50.9a | 53.3ab | 1.4 | 0.01 |
| Propionate (P), mmol/100mol | 32.5 | 28.2 | 31.3 | 33.1 | 26.1 | 31.8 | 33.4 | 30.3 | 1.5 | 0.18 |
| A:P (acetate:propionate) | 1.65 | 2.43 | 2.07 | 1.57 | 2.39 | 2.06 | 1.84 | 2.03 | 0.17 | 0.17 |
| Butyrate, mmol/100mol | 10.92 | 9.54 | 10.82 | 11.11 | 13.49 | 11.43 | 10.54 | 10.83 | 0.47 | 0.78 |
| Lactic acid, m*M* | 1.00 | 3.02 | 0.36 | 0.22 | 0.61 | 0.16 | 0.25 | 2.57 | 0.41 | 0.27 |

*Letters in each row indicate significant difference between treatments. The pH variables are a mean values for all animals by dietary treatment for the 24 h period starting at 08:00 h on the day of bacterial sample collection. The VFA and lactic acid concentrations are mean values for all animals on a dietary treatment for samples taken 4 h post-challenge.
